# Supplementary material for: Identification of novel inhibitors of the transcriptional coactivator MRTF-A for HCC therapy
Source: Mol Ther Oncol. 2024 Aug 6;32(3):200855. doi: 10.1016/j.omton.2024.200855 (PMC11387234; doi:10.1016/j.omton.2024.200855)
Supplement: Document S1. Figures S1–S5 and Tables S1–S6 [file mmc1.pdf]

OMTON, Volume *32*

Supplemental information

Identification of novel inhibitors of the transcriptional coactivator MRTF-A for HCC therapy

Miriam Jasmin Franz, Pia Wenisch, Petra Wohlleben, Laura Rupprecht, Vladimir Chubanov, Thomas Gudermann, Salla Kyheröinen, Maria Kristina Vartiainen, Markus R. Heinrich, and Susanne Muehlich

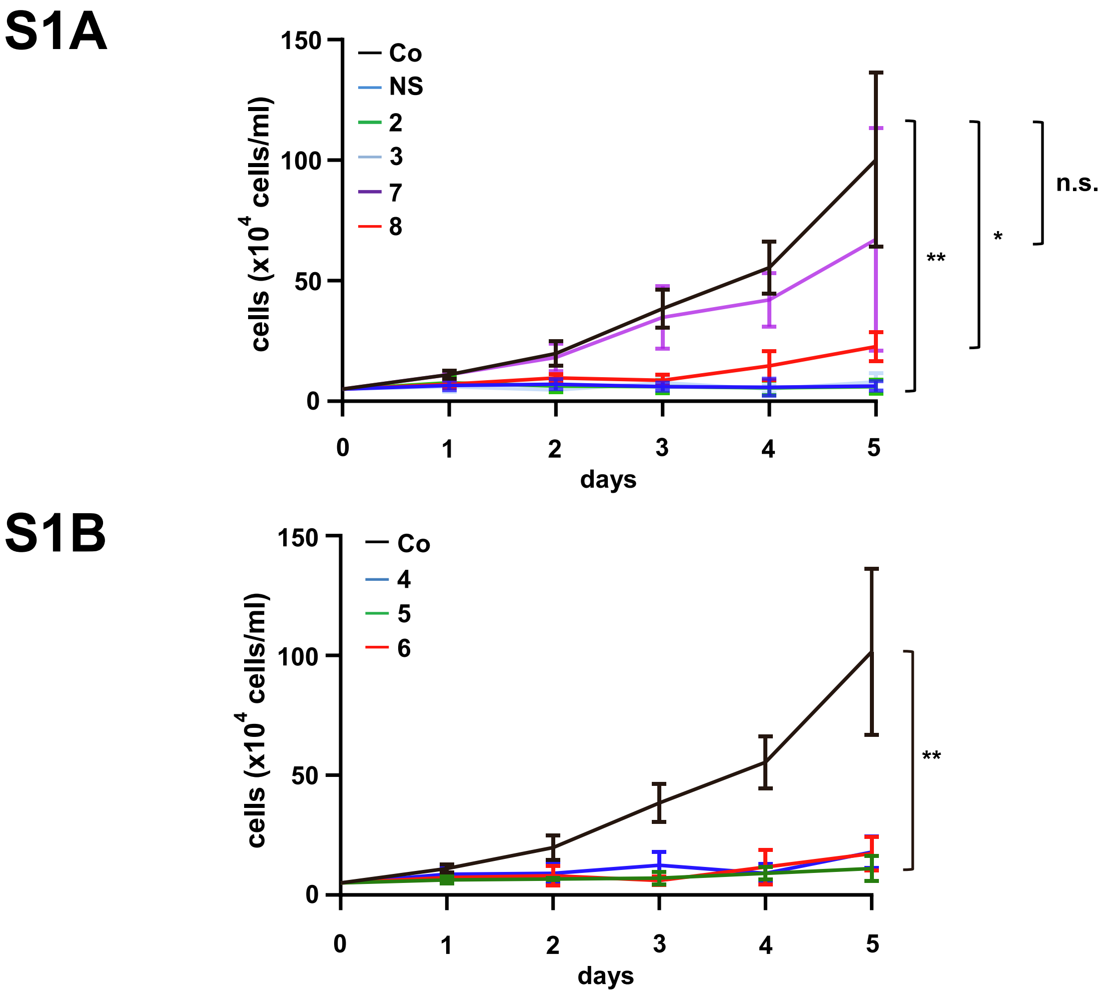

Figure S1: Inhibition of TRPM7 by NS8593 and analogues induces HCC proliferation arrest. Proliferation rates in HuH6 cells (A) treated with 30 µM NS8593, **2, 3, 7, 8** and DMSO (control, Co) and (B) inhibited with 10 µM **4**, **5**, **6** and DMSO (control, Co). Data are means ± SD (n=3).

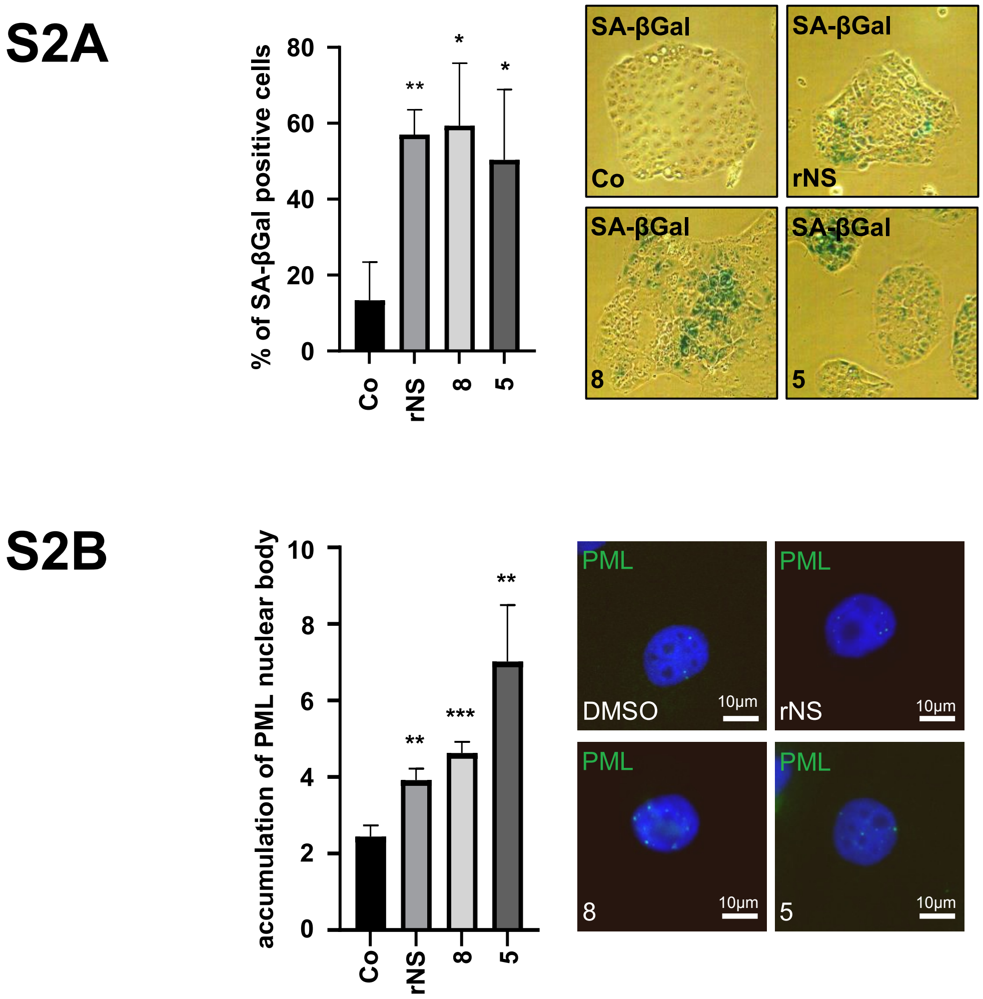

Figure S2: Inhibition of TRPM7 by NS8593 and analogues induces cellular senescence (A) Quantification of senescence-associated beta-galactosidase positive cells of HuH6 cells treated with 5 µM **5** and 30 µM rNS, **8** and DMSO (control, Co). Beta-Gal positive cells were counted in 100 cells per condition. All data are means ± SD (*n* = 3). (B) Immunofluorescence staining with anti-PML antibody and DAPI for nuclear counterstaining in HuH7 cells inhibited as in described in (A). Quantification of PML nuclear body accumulation by counting the green spots in 100 cells per condition. Data are means ± SD (n=3).

S3A

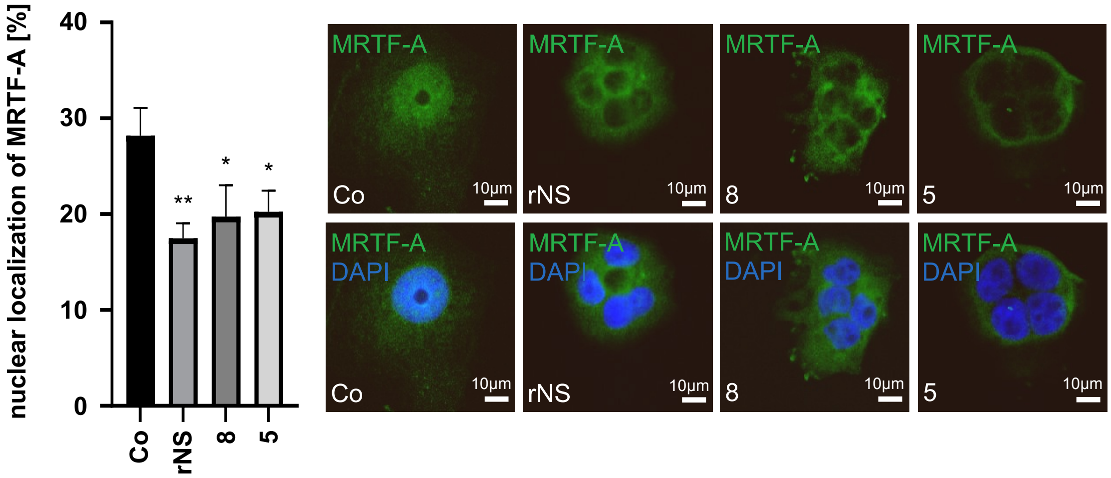

Figure S3: Enhanced MRTF-A nuclear export upon administration of rNS and compounds 5 and 8 (A) Immunofluorescence staining with anti-MRTF-A antibody and DAPI for nuclear counterstaining in HuH6 cells treated with 5 µM **5** and 30 µM rNS, **8** and DMSO (control, Co). Scale bar, 10 µm. Data are means ± SD (n=3).

S4A

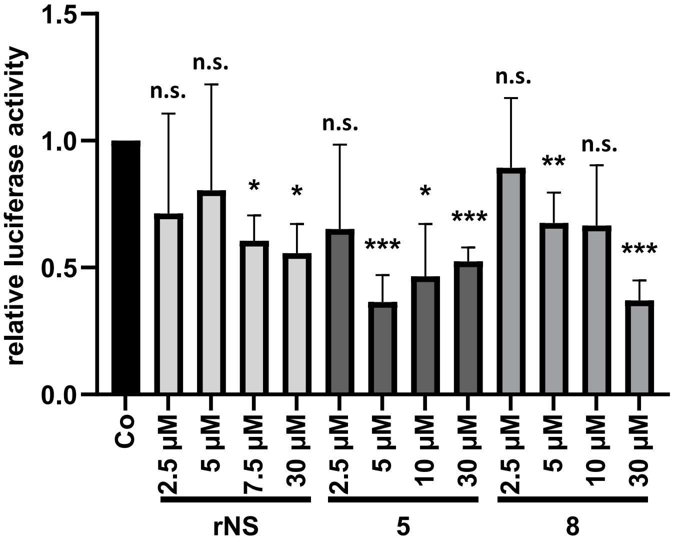

Figure S4A: Inhibitors 5 and 8 reduce SRF activity. (A) HuH7 cells expressing a SRE-dependent luciferase reporter gene (5xSRE) and a Renilla luciferase internal control (pRL-SV40P) were treated with rNS, **5**, **8** or DMSO (control, Co) as indicated and 24 h later luciferase assays performed for firefly luciferase and normalized to Renilla luciferase. Data are means ± SD (n=3).

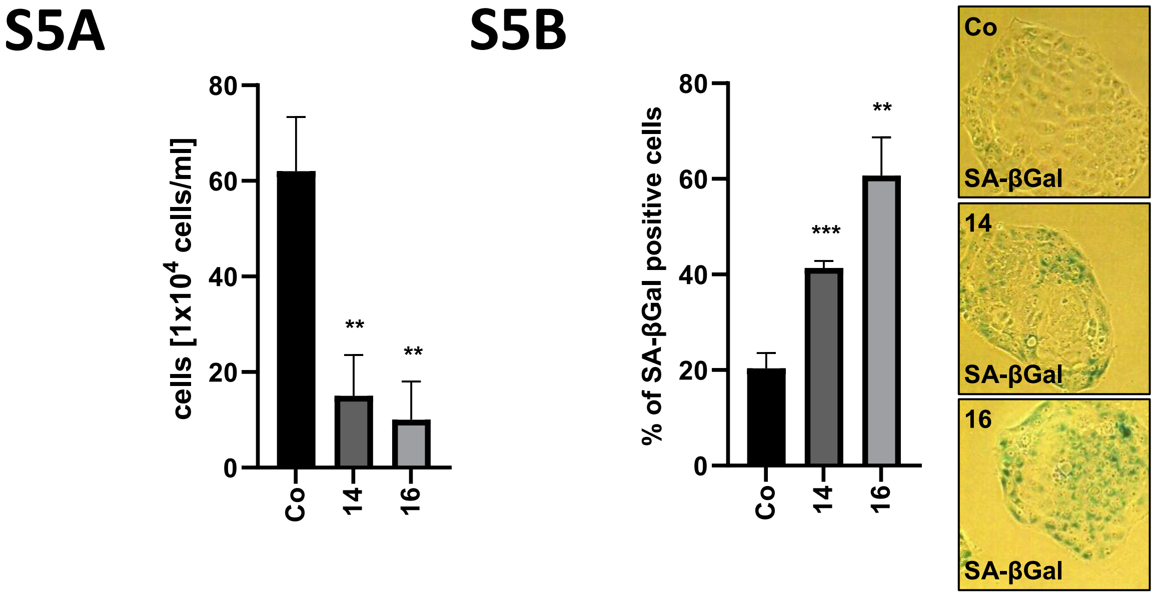

Figure S5: Novel inhibitors 14 and 16 inhibit HCC cell proliferation by inducing cellular senescence (A) Proliferation rates in HuH6 cells inhibited with 10 μM **14** and **16** and DMSO (control, Co). Data are means ± SD (n=3). (B) Quantification of senescence-associated beta-galactosidase positive HuH6 cells treated with 5 μM **14** and **16** and DMSO (control, Co). Beta-Gal positive cells were counted in 100 cells per condition. All data are means ± SD (*n* = 3).

Table S1: IC_{50} values and hill slopes for TRPM7 inhibition

derivative	IC_{50} (µM)	Hill slopes	n
NS8593	1.464	-1.432	3
1, rNS	1.761	-1.370	3
2	3.740	-1.165	3
3	5.739	-1.224	3
4	2.798	-1.123	3
5	1.093	-1.448	3
6	1.688	-1.292	3
7	63.31	-0.9579	3
8	2.817	-1.210	3

Table S2: Primary antibodies used for immunoblotting

antibody	manufacturer
anti-HSP90 (mouse)	Santa Cruz Biotechnology, Inc., Dallas, TX, USA
anti-TGFβ1 (mouse)	Santa Cruz Biotechnology, Inc., Dallas, TX, USA
anti-TSPAN5 (rabbit)	Merck, Darmstadt, Germany

Table S3: Secondary antibodies used for immunoblotting

antibody	manufacturer
anti-mouse IgG, HRP-linked antibody	Cell Signaling Technology, Danvers, MA, USA
anti-rabbit IgG, HRP-linked antibody	Cell Signaling Technology, Danvers, MA, USA

Table S4: Primary antibodies used for immunofluorescence and proximity ligation assay

antibody	manufacturer
anti-MRTF-A (mouse)	Santa Cruz Biotechnology, Inc., Dallas, TX, USA
anti-TRPM7+TRPM6 (rabbit)	Abcam, Cambridge, UK
anti-RhoA (mouse)	NewEast Bioscience, Malvern, PA, USA

Table S5: Secondary antibodies used for immunofluorescence

antibody	manufacturer
Donkey anti-mouse IgG, Alexa-488-linked antibody	Invitrogen, Karlsruhe, Germany

Table S6: p-values

Figure	Compared samples	p-values
**1D**	Co vs. 2.5 µM 5	0.0474
	Co vs. 5 µM 5	0.0016
	Co vs. 7.5 µM 5	0.0014
	Co vs. 10 µM 5	0.0014
**1E**	Co vs. NS8593	0.0016
	Co vs. 4 / 5 / 6	< 0.001

**1F**	Co vs. 7	0.0296
	Co vs. NS8593 / rNS /2 / 3 / 8	< 0.001
**2A**	Co vs. 5 µM 5	0.0073
	Co vs. 7.5 µM 5	0.0246
	Co vs. 10 µM 5	0.0034
**2B**	Co vs. 20 µM 8	0.0017
	Co vs. 30 µM 8	0.0011
**2C**	Co vs. 20 µM rNS	0.0049
	Co vs. 30 µM rNS	< 0.001
**2D**	Co vs. rNS	0.0082
	Co vs. 8	0.0014
	Co vs. 5	0.0032
**2E**	Co vs. rNS	0.0013
	Co vs. 8	< 0.001
	Co vs. 5	0.0031
**3A**	Co vs. rNS	0.031
	Co vs. 8 / 5	< 0.001
**3B**	Co vs. rNS	0.0019
	Co vs. 8 / 5	< 0.001
	rNS vs. 8	0.0427
	rNS vs. 5	0.0113
**3D**	Co vs. NS / 5	< 0.001
**4A**	Co vs. rNS / 5 / 8	< 0.001
**4B**	Co vs. 8	0.0094
	Co vs. 5	< 0.001
**4C TSPAN5**	Co vs. 5 µM 5	< 0.001
	Co vs. 7.5 µM 5	0.0326
	Co vs. 10 µM 5	0.0015
**4C TGFβ1**	Co vs. 5 µM 5	0.0075
	Co vs. 7.5 µM 5	< 0.001
	Co vs. 10 µM 5	< 0.001
**4D**	Co vs. 20 µM 8	0.012
	Co vs. 30 µM 8	0.0078
	Co vs. 40 µM 8	0.0068
	Co vs. 50 µM 8	0.0071
**5E**	Co vs. 9	0.0076
	Co vs. 10 / 12	< 0.001
**5F**	Co vs. 10	< 0.001
**6B**	Co vs. 10 / 14 / 15 / 16 / 17 / 18	< 0.001
	Co vs. 19	0.0013
	Co vs. 20	0.0012
	Co vs. 21	0.0063
	10 vs. 14 / 16	< 0.001
**6C**	Co vs. 14 / 16	< 0.001
**6D**	Co vs. 2.5µM-10µM 14	< 0.001
	Co vs. 2.5 µM 16	0.0037

	Co vs. 5μM-10μM 16	< 0.001
**6E**	Co vs. 5 μM 14	0.0458
	Co vs. 2.5 μM 16	< 0.001
	Co vs. 5 μM 16	0.0035
**7A**	Co vs. 2.5 μM 14	0.0321
	Co vs. 5 μM 14	0.0017
	Co vs. 7.5 μM 14	0.0043
	Co vs. 10 μM 14	0.0017
	Co vs. 2.5 μM 16	0.0016
	Co vs. 5μM-10μM 16	< 0.001
**7B**	Co vs. 14	0.0321
	Co vs. 16	0.0016
**7C**	Co vs. 14 / 16	< 0.001
