# Supplementary material for: Identification of novel inhibitors of the transcriptional coactivator MRTF-A for HCC therapy
Source: Mol Ther Oncol. 2024 Aug 6;32(3):200855. doi: 10.1016/j.omton.2024.200855 (PMC11387234; doi:10.1016/j.omton.2024.200855)
Supplement: Document S2. Article plus supplemental information [file mmc2.pdf]

Molecular Therapy
Oncology
Original Article

Identification of novel inhibitors of the transcriptional coactivator MRTF-A for HCC therapy

Miriam Jasmin Franz,[1,5] Pia Wenisch,[1,5] Petra Wohlleben,[1] Laura Rupprecht,[1] Vladimir Chubanov,[2] Thomas Gudermann,[2] Salla Kyheröinen,[3] Maria Kristina Vartiainen,[3] Markus R. Heinrich,[1,4] and Susanne Muehlich[1,4]

[1]Department of Chemistry and Pharmacy, Friedrich-Alexander-Universität Erlangen-Nürnberg, Nikolaus-Fiebiger-Straße 10, 91058 Erlangen, Germany; [2]Walther-Straub Institute of Pharmacology and Toxicology, Ludwig-Maximilians-Universität München, Goethestraße 33, 80336 München, Germany; [3]Institute of Biotechnology, HiLIFE, University of Helsinki, Viikinkaari 5d, 00790 Helsinki, Finland; [4]FAU NeW-Research Center for New Bioactive Compounds, Nikolaus-Fiebiger-Straße 10, 91058 Erlangen, Germany

Myocardin-related transcription factor A (MRTF-A) is a coactivator of serum response factor (SRF), which regulates the expression of genes involved in cell proliferation, migration, and differentiation and has been implicated in hepatocellular carcinoma (HCC) progression. We recently established inhibition of the transcriptional activity of MRTF-A by NS8593 as a novel therapeutic approach for HCC therapy. NS8593 is a negative gating modulator of the transient receptor potential cation channel TRPM7. In this report, we identify an aminobenzimidazole that is highly potent in inhibiting TRPM7 and its interaction with RhoA, leading to decreased SRF transcriptional activity and enhanced nuclear export of MRTF-A, as determined by fluorescence loss in photobleaching (FLIP). This resulted in reduced expression of the MRTF/SRF target genes transforming growth factor β1 (TGF-β1) and tetraspanin 5 (TSPAN5), senescence induction, and growth arrest in HCC cells. Replacement of the tetraline core by a 3-aminophenyl substructure yielded inhibitor 10 with higher potency than inhibitor 5, and further structural modifications yielded highly potent inhibitors of SRF activity, 14 and 16. Both compounds were capable of inhibiting cell proliferation and inducing senescence in HCC cells with improved efficacy compared to NS8593. These inhibitors represent valuable tools for understanding the molecular basis of drug development targeting TRPM7 and MRTFs.

INTRODUCTION

Myocardin-related transcription factor A (MRTF-A) is a coactivator of serum response factor (SRF), which regulates the expression of genes involved in cell proliferation, migration, and differentiation and plays an important role in hepatocellular carcinoma (HCC) growth. HCC is the second-leading cause of cancer-related deaths worldwide.[1] An inflammatory environment resulting in liver fibrosis and cirrhosis is regarded as a pre-neoplastic stage, and 80%–90% of HCC patients have been diagnosed previously with liver cirrhosis.[2,3] Due to the lack of therapeutic options for HCC, surgery often remains the option of choice, and the 5-year survival rate is less than 15%.[4] Therefore, there is an urgent need to identify novel drug targets and inhibitors for HCC therapy.

We have previously demonstrated that targeting MRTF-A inhibits HCC xenograft growth.[5] Conditional expression of constitutively active SRF in hepatocytes triggers HCC formation, accompanied by increased expression of MRTF/SRF target genes such as Myoferlin.[6,7] Global deletion of MRTF-A in a murine high-fat diet-induced liver injury model has been shown to decrease liver fibrosis.[8] Anti-fibrotic effects of MRTF-A blockade have also been observed upon administration of the small molecule CCG-203971 in hepatic stellate cells *in vitro* and *in vivo*.[9] Therefore, MRTF-A represents a promising target for novel therapeutic interventions for HCC therapy.

We have demonstrated previously that targeting MRTF-A inhibits HCC xenograft growth by inducing oncogene-induced senescence (OIS).[5] OIS has emerged as a tumor-suppressive mechanism and has gained importance for pharmacological intervention in HCC therapy.[10]

We recently established senescence induction upon inhibition of the nuclear localization and transcriptional activity of MRTF-A by the

Received 1 May 2024; accepted 2 August 2024;
https://doi.org/10.1016/j.omton.2024.200855.

[5]These authors contributed equally

Correspondence: Markus R. Heinrich, Department of Chemistry and Pharmacy, Friedrich-Alexander-Universität Erlangen-Nürnberg, Nikolaus-Fiebiger-Str. 10, 91058 Erlangen, Germany.
E-mail: markus.heinrich@fau.de
Correspondence: Susanne Muehlich, Department of Chemistry and Pharmacy, Friedrich-Alexander-Universität Erlangen-Nürnberg, Nikolaus-Fiebiger-Str. 10, 91058 Erlangen, Germany.
E-mail: susanne.muehlich@fau.de

Published by Elsevier Inc. on behalf of The American Society of Gene and Cell Therapy.
This is an open access article under the CC BY license (http://creativecommons.org/licenses/by/4.0/).

negative gating modulator NS8593 of the transient receptor potential cation channel TRPM7 as a novel therapeutic approach for HCC therapy.[11] TRPM7 is a bifunctional protein comprising a transmembrane ion channel prevalently gating divalent cations as well as a cytosolic serine/threonine protein kinase.[12–16] TRPM7 is essential for cell proliferation and cell growth[17] and has been associated with a multitude of cancers, such as ovarian, pancreatic, and colorectal cancer.[18] Since TRPM7 is the first druggable target in HCC, the development of novel NS8593-like compounds that are able to inhibit the TRPM7 channel, increase MRTF-A nuclear export, and thereby decrease SRF transcriptional activity is of utmost importance.

Besides the TRPM7 channel,[19] NS8593 modulates small-conductance Ca^{2+}-activated K^{+} (SK) channels.[19] Despite their negligible effect on MRTF-A localization and MRTF-A transcriptional activity,[11] SK channel blockade can cause undesired adverse effects such as tremors, mediated by actions in the CNS.[20,21] Therefore, in order to avoid these possible adverse effects, the SK channel affinity must also to be taken in account for the design of structurally close analogs of NS8593.

In this work, we now present the results of a structure-activity relationship study on NS8593 derivatives that revealed that aminobenzimidazole **5** promoted nuclear export of MRTF-A, decreased SRF transcriptional activity and target gene expression, and improved selectivity for inhibition of the TRPM7 channel and subsequent TRPM7/RhoA interactions.

In addition, aminobenzimidazole **8** exhibited attenuated SK channel affinity relative to NS8593 and also inhibited TRPM7/RhoA interactions, MRTF-A nuclear localization, SRF activity, and target gene expression. Both compounds were capable of inhibiting cell proliferation and senescence induction with improved efficacy than NS8593 in HCC cells.

Based on these results, we replaced the tetraline core of compounds **5** and **8** by 2- or 3-aminophenyl substructures to obtain inhibitors **9**–**21**. These structural modifications afforded the highly potent MRTF/SRF inhibitors **10**, **14**, and **16** for potential future HCC therapy.

RESULTS

Synthesis of target compounds 1–8 and determination of concentration dependencies for inhibition of TRPM7 and HCC cell proliferation

We have reported recently that the TRPM7 inhibitor NS8593 abolishes HCC growth.[11] In order to improve the selectivity and potency of NS8593 while attenuating potential adverse effects of NS8593 mediated by SK channel blockade, we sought to generate structurally close analogs of NS8593 differing in their affinity for the TRPM7 and SK channels. Within the initial structure-activity relationship study, we focused on exploring the effects of substituents on the tetraline subunit of the lead compound NS8593 (Figure 1A). While compound **1** was prepared as a racemic form of NS8593 (rNS) to serve as control in the biological evaluations, the aminotetralines **2**–**5** bear varying substituents in positions 5–8 of the 1-aminotetraline. Moreover, two heterocyclic derivatives, **7** and **8**, incorporating either a pyridine or a thiophene substructure, respectively, were synthesized (Figure 1A).

For the preparation of the target compounds **1**–**8**, the eight respective ketones were coupled to 2-aminobenzimidazole by reductive amination. Titanium(IV) isopropoxide and sodium triacetoxyborohydride were used as reagents to give the desired N-functionalized aminobenzimidazoles **1**–**8** in isolated yields ranging from 34% to 58% (Figure 1A). We first determined the half-maximal inhibitory concentration (IC_{50}) for NS8593 and **1**–**8** using Ca^{2+} imaging. Among all structural variants, compound **5** exhibited the lowest IC_{50} of 1.093 μM as compared to NS8593 with an IC_{50} of 1.464 μM (Figure 1B; Table S1). Notably, and due to their importance as off targets mediating potential side effects, the aminobenzimidazoles **1**–**8** were also chosen with regard to known data on the inhibition of SK3 channels (Figure 1C).[22]

Functional characterization of HCC cell proliferation demonstrated that inhibitor **7**, exhibiting the highest IC_{50} of 63.31 μM for TRPM7, decreased cell proliferation only marginally, whereas all others caused growth arrest in HuH7 and HuH6 cells (Figures 1D, 1E, S1A, and S1B). Notably, compound **5** significantly reduced HuH7 cell growth already at 2.5 μM concentration (Figure 1F). Taken together, we demonstrated, within this first series of compounds, a clear correlation between inhibition of TRPM7 activity and HCC cell proliferation.

Inhibition of TRPM7 by NS8593 and analogs induces cellular senescence

We next sought to investigate whether senescence induction is the molecular mechanism underlying the observed proliferation arrest in HCC cells treated with NS8593 and analogs. Senescence-associated β-galactosidase (SA-β-Gal) staining revealed that 5 μM of compound **5** was sufficient to significantly increase the percentage of SA-ß-Gal-positive HuH7 and HuH6 cells (Figures 2A and S2A). In contrast, 20 μM compound **8** and rNS were required to significantly induce the senescence response in HuH7 and HuH6 cells (Figures 2B, 2C, and S2A). We also detected an enhanced accumulation of promyelocytic leukemia (PML) bodies in HuH7 and HuH6 cells, indicative of OIS by immunofluorescence staining (Figures 2D and S2B). We next tested whether compound **5** also exerts other anti-tumorigenic effects on invasion. Indeed, we found that compounds **5** and **8** and rNS strongly impaired the penetration of HuH7 cells into Matrigel (Figure 2E). Taken together, these results show that compound **5** exerts anti-invasive and anti-proliferative effects, mediated by inducing OIS at 6-fold lower concentrations than NS8593.

Impairment of TRPM7-RhoA interaction leads to enhanced MRTF-A nuclear export We next examined the molecular mechanism underlying the improved selectivity for inhibition of the TRPM7 channel by compounds **5** and **8** and its consequences on the TRPM7-MRTF-A axis. Because our previous results suggested that TRPM7 regulates MRTF-A transcriptional activity by impinging on the RhoA-TRPM7 interaction,[11] we next assessed the RhoA-TRPM7

A

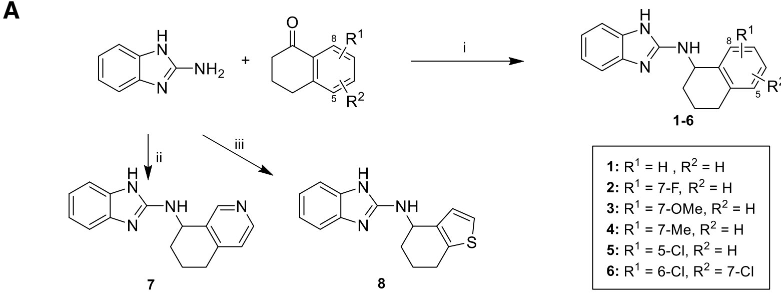

B

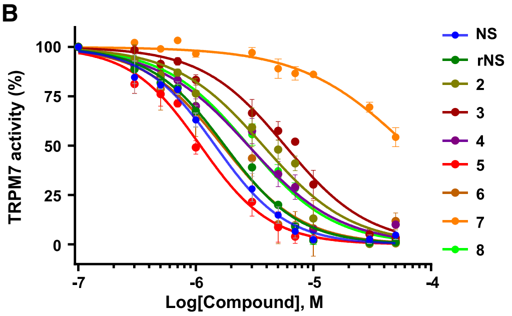

C

derivative	TRPM7 affinity	SK3 affinity
1, rNS	=NS	=NS
2	<NS	>NS
3	<NS	=NS
4	<NS	>NS
5	>NS	=NS
6	=NS	>NS
7	<NS	<NS
8	<NS	<NS

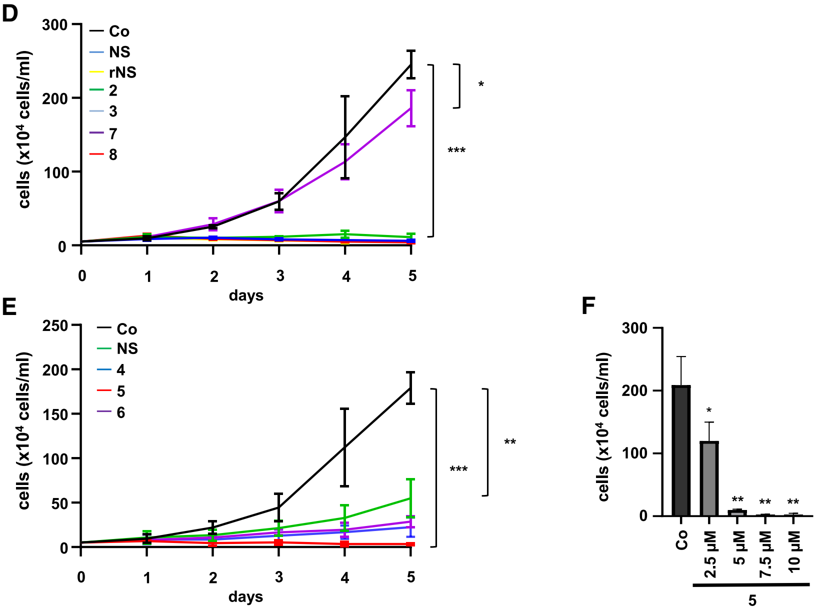

Figure 1. Synthesis of target compounds 1–8 and determination of concentration dependencies for inhibition of TRPM7 and cell proliferation

(A) Synthesis of target compounds **1**–**8**. Reagents and conditions: (i) titanium(IV) isopropoxide, $NaB(O_2CCH_3)_3$, THF, rt, N_2, 38%–58%; (ii) 6,7-dihydroisoquinolin-8(5H)-one, titanium(IV) isopropoxide, $NaB(O_2CCH_3)_3$, THF, rt, N_2, 34%; (iii) 4-keto-4,5,6,7-tetrahydrothianaphthene, titanium(IV) isopropoxide, $NaB(O_2CCH_3)_3$, THF, rt, N_2, 41%. (B) Concentration dependencies for inhibition of TRPM7 by NS8593 (NS), rNS (**1**) and **2**–**8** generated by the Ca^{2+} influx assay. Curves through the points (mean ± SEM) are logistic equation fits. The values of IC_{50} and hill slopes are provided in Table S1. (C) TRPM7 and SK3 channel affinities (taken from Sørensen et al.[22]) of NS derivatives compared to NS. (D and E) Proliferation rates in HuH7 cells treated with (D) 30 μM NS, rNS, **2**, **3**, **7**, **8**, and DMSO as a control and (E) 10 μM NS, **4**, **5**, **6**, and DMSO. Data are means ± SD (n = 3); **p* < 0.05, ***p* < 0.01, ****p* < 0.001. (F) Concentration dependencies for inhibition of HuH7 cell proliferation upon administration of 2.5, 5, 7.5, and 10 μM compound **5**. Data are means ± SD (n = 3); **p* < 0.05, ***p* < 0.01.

interaction and MRTF-A localization upon TRPM7 blockade using compounds **5** and **8.** 5 μM compound **5** inhibited RhoA-TRPM7 interaction to an extent approaching that of 30 μM compound **8** and the lead rNS, as determined by proximity ligation assays (Figure 3A). The strongest effects of compounds **5** and **8** were observed on MRTF-A nuclear localization in HuH7 and HuH6 cells. MRTF-A nuclear localization was reduced to 12% upon treatment with 30 μM compound **8** and 5 μM compound **5** (Figures 3B and S3A). Compared to the lead rNS, achieving an inhibition of MRTF-A nuclear localization by 50%, a significant reduction in nuclear localization was observed for compounds **8** and **5** (Figure 3B).

To lend further credence to the concept that the improved selectivity for inhibition of the TRPM7 channel promotes nuclear export of MRTF-A, we studied nuclear export of MRTF-A upon treatment with NS8593 and compound **5** with the fluorescence loss in photobleaching (FLIP) assay. For this approach, we continuously bleached the cytoplasm and measured the loss of nuclear fluorescence due to the nuclear export of MRTF-A-GFP. After 80 s of bleaching in HuH7 cells treated with compound **5** and NS8593, there was less MRTF-A-GFP fluorescence remaining in the nucleus upon treatment with compound **5** as compared to the control cells (Figures 3C and 3D). It should be noted that the effect of compound **5** on nuclear export was even more pronounced because a much lower concentration (5 μM) was used than for NS8593 (30 μM). Our results clearly indicate increased nuclear export of MRTF-A after treatment with compound **5** and warrants further investigation how the lower portion of nuclear MRTF-A impacts SRF transcriptional activation and the expression of MRTF/SRF target genes.

Novel inhibitor 5 reduces SRF activity and MRTF/SRF target gene expression

In order to investigate whether the lower portion of MRTF-A remaining in the nucleus upon treatment with compound **5** reflects the availability of MRTF-A to coactivate SRF, we performed SRF reporter gene assays. Expression of a 5×SRE-luciferase reporter gene led to a strong decrease in SRF activity upon administration of rNS and compounds **5** and **8** (Figure 4A). Titration experiments allowed us to examine the

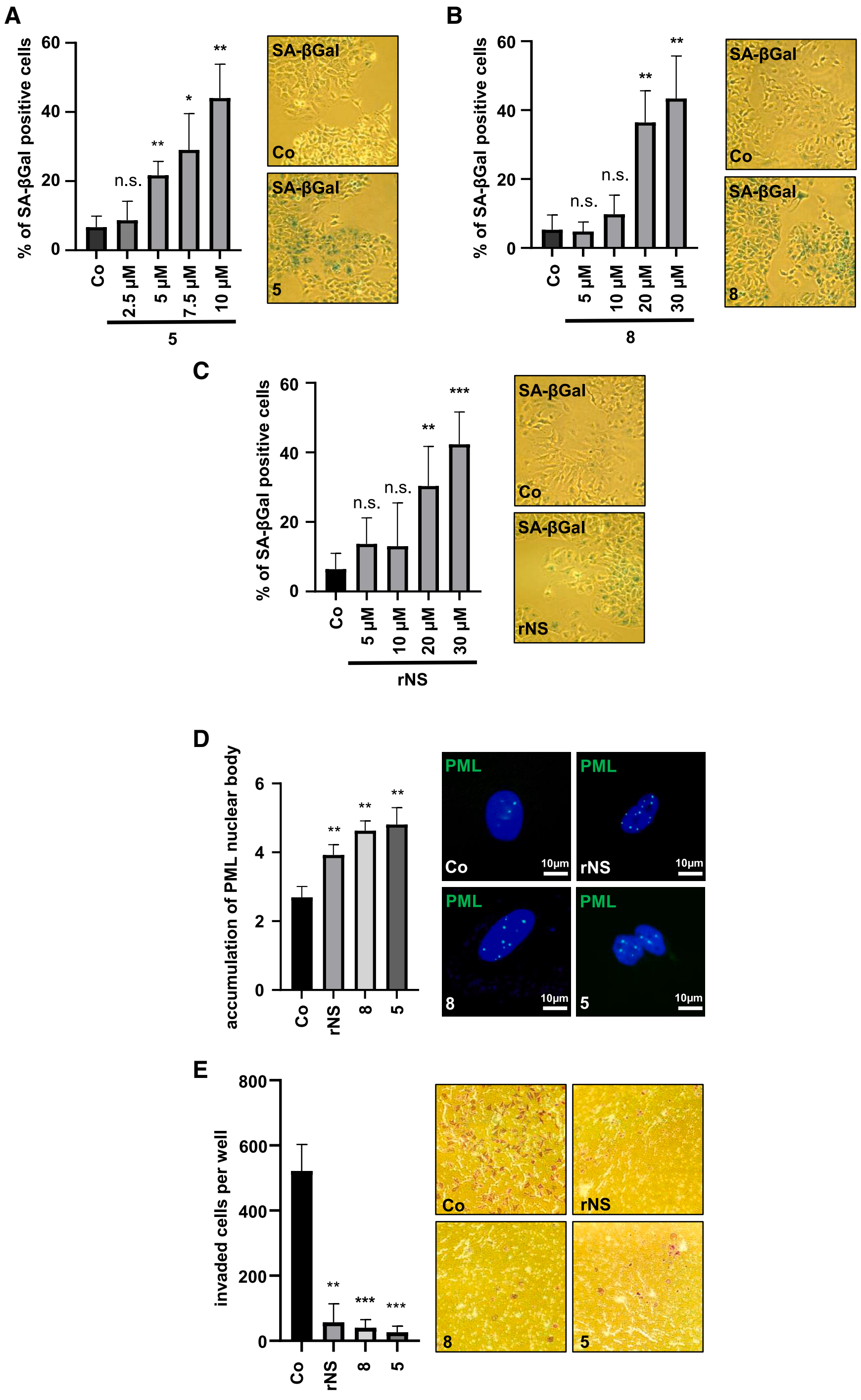

Figure 2. Inhibition of TRPM7 by NS and analogs induces cellular senescence

(A–C) Quantification of SA-β-Gal-positive HuH7 cells treated with compounds **5** (A), **8** (B), and rNS (C) as indicated and DMSO as a control (left). β-Gal-positive cells were counted in 100 cells per condition. All data are means ± SD (n = 3); *$p < 0.05$, **$p < 0.01$, ***$p < 0.001$, n.s. not significant. Shown are representative pictures of SA-β-gal staining with **5** (5 μM), rNS, and **8** (30 μM) (right). (D) Immunofluorescence staining with anti-PML antibody and DAPI for nuclear counterstaining in HuH7 cells treated with inhibitor of **5** (5 μM), **8**, and rNS (30 μM) and DMSO as a control. Shown is quantification of PML nuclear body accumulation in 100 cells per condition. Scale bar, 10 μm. Data are means ± SD (n = 3); **$p < 0.01$. (E) HuH7 cells were treated as described in (D) and subjected to Matrigel invasion assay chambers, and after 20 h, invaded cells were stained by crystal violet and counted. All data are means ± SD (n = 3); **$p < 0.01$, ***$p < 0.001$.

effect of up to 6-fold lower concentrations (5 μM) of rNS and compounds **5** and **8** on SRF activity (Figure S4A). There was a 60% reduction in SRF activity upon treatment with 5 μM compound **5** and a 30% reduction upon treatment with 5 μM compound **8** (Figures 4B and S4A). These findings showed that compound **5** is significantly more effective than compound **8** as well as the lead structure rNS in inhibiting SRF transcriptional activity. The difference between luciferase activity in HuH7 cells treated with 5 μM compound **5** and 5 μM compound **8** was statistically significant (Figure 4B). We hypothesized that a critical threshold for inhibition of the SRE must be achieved before effects on MRTF/SRF target genes are detectable. We therefore assessed the protein levels of the MRTF/SRF target genes transforming growth factor β1 (TGF-β1) and TSPAN5, which play an important role in HCC proliferation and senescence.[7,23] Indeed, treatment with 5 μM compound **5** almost completely abolished TGF-β1 and TSPAN5 expression (Figure 4C), whereas 20 μM compound **8** was required to significantly reduce TSPAN5 expression (Figure 4D).

Together, these findings suggest that compound **5** has the strongest effect on SRF activity and may serve as a template for constructing novel improved inhibitors targeting the TRPM7-SRF axis.

Constructing novel inhibitors of MRTF/SRF activity based on compound 5

The improved properties regarding TRPM7-mediated inhibition of SRF activity by aminotetraline **5** is in agreement with the recently published cryoelectron microscopy (cryo-EM) structure of NS8593 bound to TRPM7.[24] As shown in Figure 5A, a substitution of the tetraline core of NS8593 is more likely to be tolerated in direction A than in direction B (Figure 5B). This corresponds to the observation that inhibitor **5** (substituted in direction A) displays much higher activity than inhibitors **2**–**4** and **6** with substituents pointing in direction B.

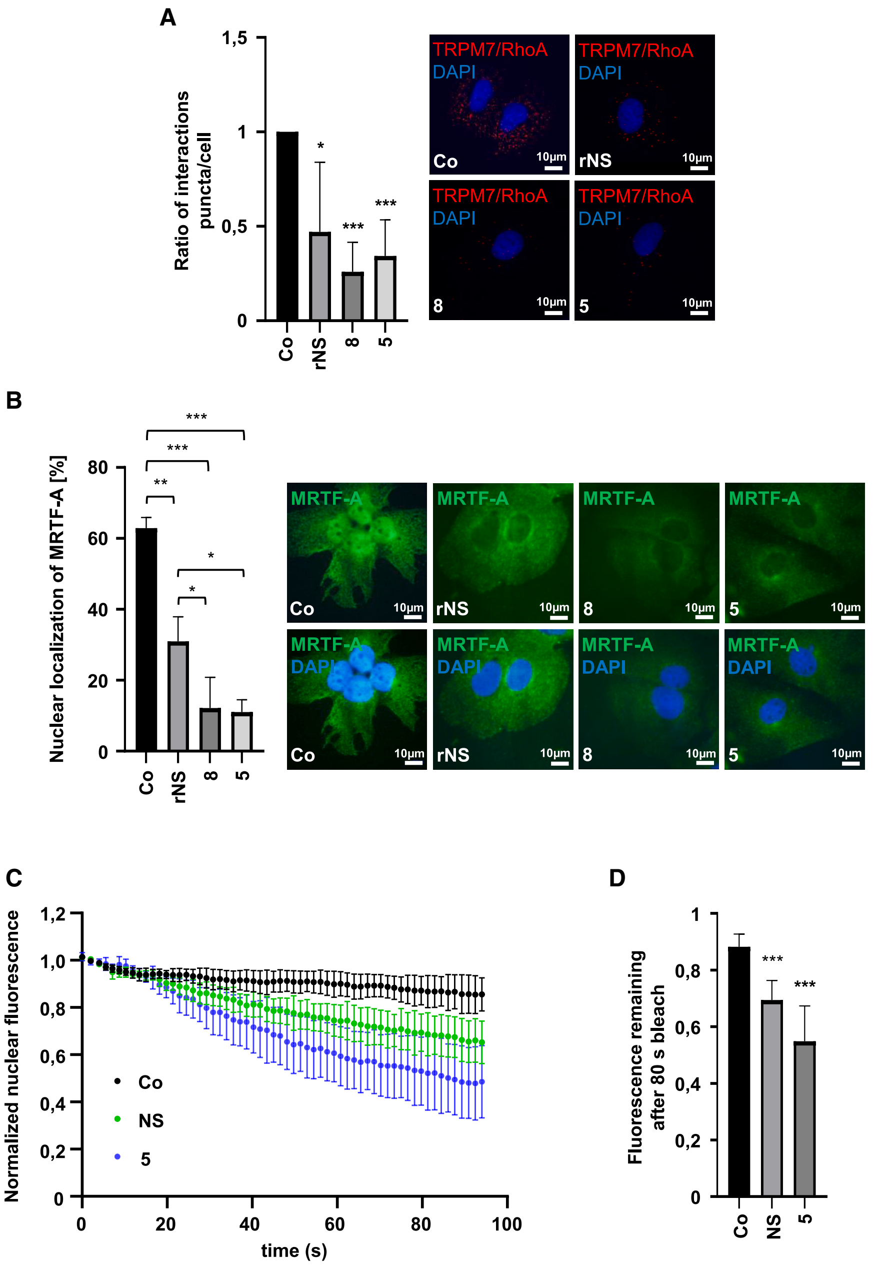

Figure 3. Impairment of TRPM7/RhoA interaction leads to enhanced MRTF-A nuclear export

(A) Immunofluorescence analysis and quantification of proximity ligation assay (PLA) for endogenous TRPM7 and RhoA in HuH7 cells treated for 6 h with compounds **5** (5 μM) and **8** and rNS (30 μM). Scale bar, 10 μm. PLA signals were counted in 15 cells per condition. Data are means ± SD (n = 3); *p < 0.05, ***p < 0.001. (B) Immunofluorescence staining with anti-MRTF-A antibody and DAPI for nuclear counterstaining in HuH7 cells treated as above. Scale bar, 10 μm. Data are means ± SD (n = 3); *p < 0.05, **p < 0.01, ***p < 0.001. (C) Fluorescence loss curves from the FLIP assay, representing nuclear export of HuH7 cells treated with compound **5** (5 μM) and NS (30 μM). Data are mean (N ≥ 8 cells per condition), normalized to pre-bleach ±SD. (D) Fluorescence remaining in the nucleus after 80 s in the FLIP assay. Data were evaluated with a Mann-Whitney test; ***p < 0.001.

On this basis, we assumed that a replacement of the tetraline core by a biphenyl unit should show an orientation toward direction A, which can be achieved by the attachment of a 3-aminobipenyl comprising ring system C (Figure 5C). A 2-aminobiphenyl substructure incorporating ring D, and pointing in direction B, should instead show much lower activity. As summarized in Figure 5D, the 2-aminobiphenyl substructure is present in compounds **9**, **11**, **12**, and **13**, whereas the 3-aminobiphenyl unit is a substructure compound **10**. The biphenyl derivatives **9**–**13**, shown in Figure 5D, as well as those summarized in Figure 6A (compounds **14**–**21**, see below) were accessible from 2-chlorobenzimidazole and the respective 2- or 3-aminobiphenyl in good to high yields using microwave irradiation. Indeed, the first functional comparison of inhibitors **9**–**13** in a luciferase assay was in agreement with our assumption of a preferential substitution in direction A (Figure 5C). While SRF activity was decreased to 25% upon incubation of HuH7 cells with substance **10**, SRF reporter gene activity remained largely unchanged upon treatment with compounds **9**, **11**, and **13** (Figure 5E). We also studied the localization of MRTF-A in response to inhibitor **10**. Endogenous MRTF-A was predominantly localized in the nucleus in HuH7 cells and was cytoplasmic following incubation with inhibitor **10** (Figure 5F). These findings suggest that **10** is a promising lead compound for the development of novel pharmacologic tools to disrupt transcriptional responses of the TRPM7-RhoA-MRTF-A axis.

Constructing novel inhibitors of MRTF/SRF activity based on compound 10

With the aim of further optimization, we next undertook structural modifications on the 3-aminobiphenyl substructure of compound **10**. Similar to the synthesis route described in Figure 5D above, the new analogs **14**–**21** could be obtained through nucleophilic aromatic substitution of 2-chlorobenzimidazole with the respective 3-aminobiphenyl (Figure 6A).

Among these, compounds **14** and **16** significantly improved inhibitory effects on HCC proliferation as compared to inhibitor **10** (Figure 6B). Both compounds provoked a proliferation arrest reminiscent of cellular senescence (Figures 6C, S5A, and S5B). Titration experiments using 10, 7.5, 5, and 2.5 μM concentrations demonstrated

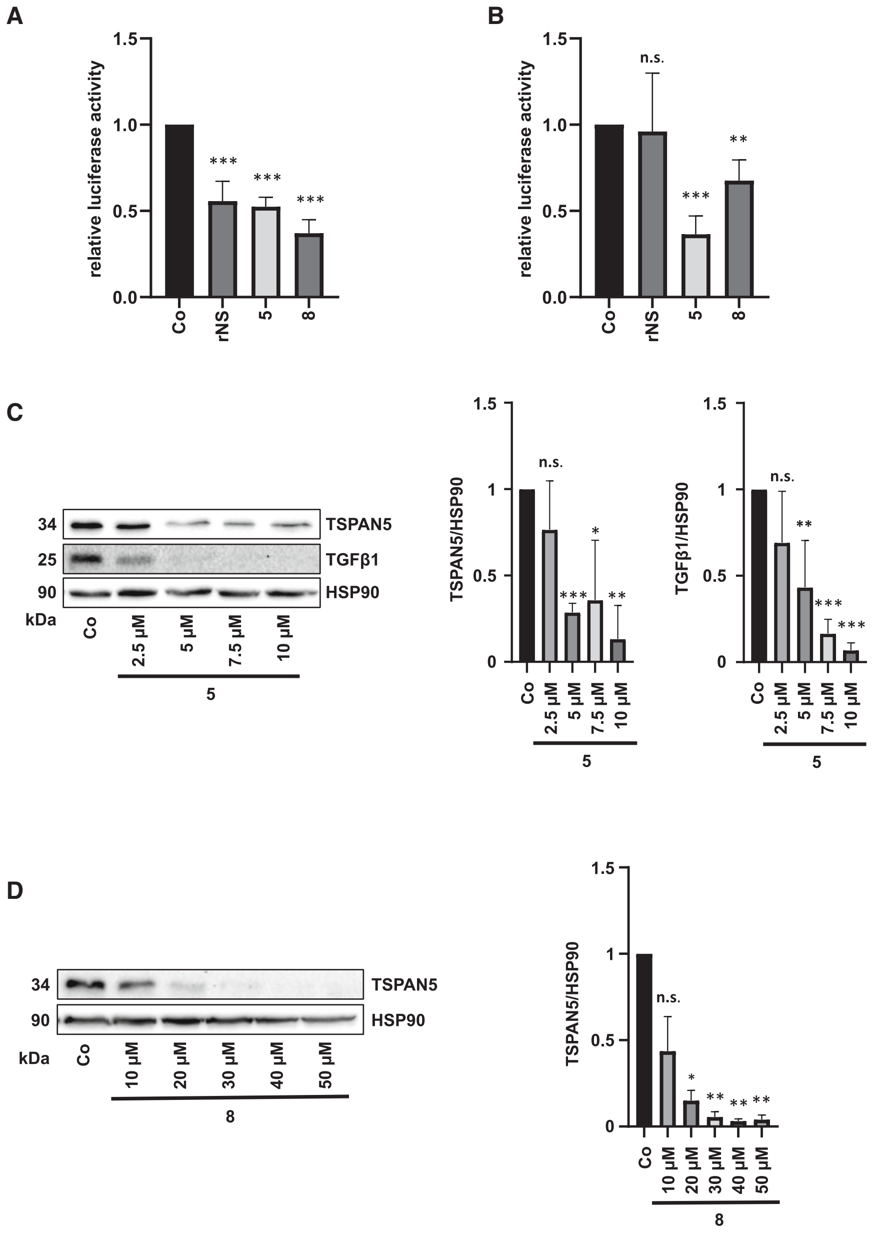

Figure 4. Novel inhibitor 5 reduces SRF activity and MRTF/SRF target gene expression

(A) HuH7 cells expressing an SRE-dependent luciferase reporter gene (5×SRE) and a *Renilla* luciferase internal control (pRL-SV40P) were treated with 30 μM **5**, **8**, rNS, or DMSO as a control, and 24 h later, luciferase assays were performed for firefly luciferase and normalized to *Renilla* luciferase. Data are means ± SD (n = 3); ***p < 0.001. (B) HuH7 cells were treated as above. A concentration of 5 μM was used. Data are means ± SD (n = 3); **p < 0.01, ***p < 0.001, n.s. not significant. (C) Immunoblotting (left) for TSPAN5, TGF-β1, and HSP90 as a loading control and quantification (right) of HuH7 cells treated with compound **5** as indicated. Data are means ± SD (n = 3); *p < 0.05, **p < 0.01, ***p < 0.001, n.s. not significant. (D) Immunoblotting (left) for TSPAN5 and HSP90 as a loading control and quantification (right) of lysates of HuH7 cells treated with **8** and DMSO as a control group with anti-TSPAN5 and anti-HSP90 antibody. Data are means ± SD (n = 3); *p < 0.05, **p < 0.01, n.s. not significant.

that 2.5 μM of compound **14** and **16** was sufficient to cease HCC cell proliferation (Figure 6D). Compound **16** significantly induced cellular senescence with as little as 2.5 μM, as determined by SA-β-Gal staining (Figure 6E). Similar results were obtained with PML staining (Figure 6F). Together, these results show that novel compounds **14** and **16** are superior in mediating HCC growth arrest and senescence induction.

Compounds 14 and 16 inhibit MRTF/SRF activity

Given the effect of the novel compounds **14** and **16** on HCC proliferation and senescence induction, they should resemble their effects on SRF activity and MRTF-A localization. We therefore performed SRF reporter gene and immunofluorescence assays.

Indeed, a significant reduction of SRF activity already occurred at 2.5 μM concentrations (Figures 7A and 7B). Likewise, a significant reduction in nuclear localization of MRTF-A already occurred at 2.5 μM concentration (Figure 7C). Thus, among our novel inhibitors, compounds **14** and **16** had the strongest effect on SRF activity and MRTF-A nuclear localization.

DISCUSSION

Despite HCC being the second-leading cause of cancer mortality in the world, little progress has been made in HCC therapy. Molecularly targeted therapies and novel inhibitors for HCC therapy are therefore urgently needed.

We recently identified the negative gating modulator NS8593 of the transient receptor potential cation channel TRPM7 as a novel inhibitor of MRTF/SRF-mediated gene transcription.[11] As shown in the model in Figure 7D, TRPM7 blockade by NS8593 inhibits RhoA activation via TRPM7's kinase domain. Inhibition of RhoA then leads to disassembly of actin stress fibers and an increase in G-actin levels. Since G-actin directly binds to MRTF-A's N-terminal RPEL motifs, the increase in G-actin levels leads to enhanced nuclear export of MRTF-A. This reduces the availability of MRTF-A to transactivate SRF, leading to HCC growth arrest and induction of senescence. The goal of our study was to improve the selectivity and potency of NS8593 to provide novel MRTF inhibitors for HCC therapy and new insights into the TRPM7-MRTF-A-SRF signaling pathway for therapeutic intervention.

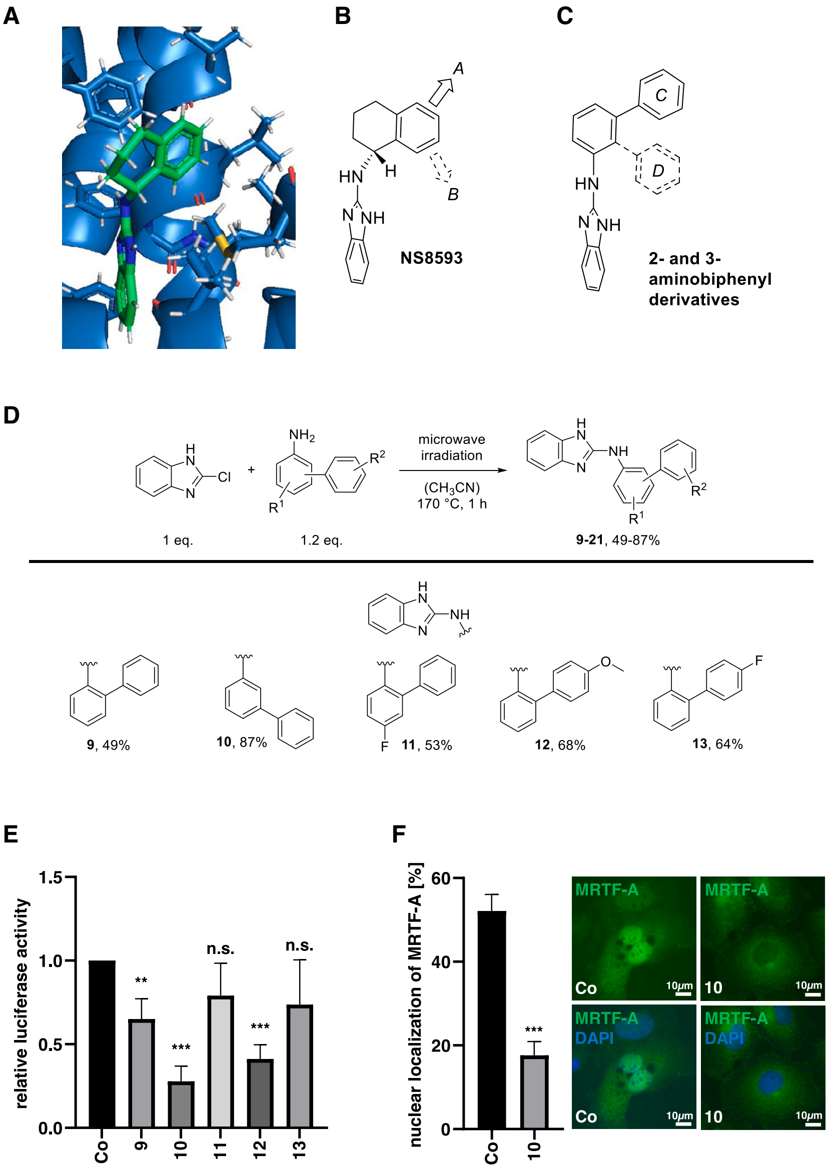

Figure 5. Constructing novel inhibitors of MRTF/SRF activity based on compound 5

(A) Docking of NS using the cryo-EM structure of TRPM7 (PDB: 8SIA)[24] and AutoDockVina. (B) Structure of NS and orientations A and B for substitution on the tetraline core. (C) Aminobiphenyl derivatives incorporating a 2- or 3-aminobiphenyl substructure (attachment of rings D or C). (D) The biphenyl inhibitors **9–21** were synthesized from the corresponding 2- or 3-aminobiphenyls, which were prepared previously by either a radical Gomberg-Bachmann reaction[25] or a Suzuki cross-coupling reaction.[26,27] The inhibitors **9–21** were then obtained through a nucleophilic aromatic substitution of 2-chlorobenzimidazole with the respective aminobiphenyl applying microwave irradiation. The desired target compounds **9–21** were isolated in yields ranging from 49%–87%.[22] (E) HuH7 cells expressing 5xSRE and pRL-SV40P were treated with 10 μM **9–13** or DMSO as a control, and 24 h later, luciferase assays were performed for firefly luciferase and normalized to *Renilla* luciferase. Data are means ± SD (n = 3); $^{**}p < 0.01$, $^{***}p < 0.001$, n.s. not significant. (F) Immunofluorescence staining with anti-MRTF-A antibody and DAPI for nuclear counterstaining in HuH7 cells treated for 18 h with 5 μM **10** and DMSO as a control group. Scale bar, 10 μm. Data are means ± SD (n = 3); $^{***}p < 0.001$.

Here, we identified the novel NS-like compound **5** by an initial structure-activity relationship study with improved potency for inhibition of TRPM7 and its interaction with RhoA, leading to decreased SRF transcriptional activity.

One of the most intriguing aspects gleaned from this study is the observation that compound **5** significantly reduced MRTF-A nuclear localization. The simplest explanation is that the rate of nuclear export was increased. Indeed, photobleaching experiments confirmed that MRTF-A was exported more rapidly upon treatment with compound **5** than NS8593. Since MRTF-A phosphorylation and G-actin binding is required for its nuclear export,[28,29] one possibility is that the nuclear G-actin levels are high in the nuclei treated with compound **5**, so that they bind MRTF-A sufficiently to induce its nuclear export. The cellular balance of G-actin in the nuclear and cytoplasmic compartments is controlled by active nucleocytoplasmic shuttling, and aberrant nuclear actin abundance has been observed in cancer.[30] Another signaling pathway that could play a role in enhanced nuclear export of MRTF-A is the Ras/mitogen-activated kinase pathway, which is also activated upon administration of NS8593.[11] In several other publications, modulation of RhoA activity and nuclear actin levels has been linked to MRTF/SRF target gene expression; e.g., in response to CCG-1423 or CCG-203971.[9,31]

However, TRPM7 represents the first druggable target at the plasma membrane to prevent MRTF-A function as a transcriptional coactivator linking RhoA and SRF activation.[11] TRPM7 is a constitutive active channel tightly regulated by Mg^{2+}, Mg-ATP, and phosphatidylinositol-4,5-bisphosphate with a C-terminal protein kinase domain, which phosphorylates and activates RhoA and several other protein substrates such as Smad2.[11,12,32,33] Our previous study provided information for the design of small molecules targeting the TRPM7-RhoA-MRTF-axis.[11] Given the fact that RhoGTPases are globular structures without useful grooves and pockets on their surface for high-affinity chemical binding, they are not considered as traditional druggable targets.[34] Therefore, TRPM7 serves as an effective target because it results in suppression of downstream RhoA-MRTF signaling.

Our data is in agreement with the recently discovered cryo-EM structure of TRPM7,[24] according to which inhibitor **5** substituted at the

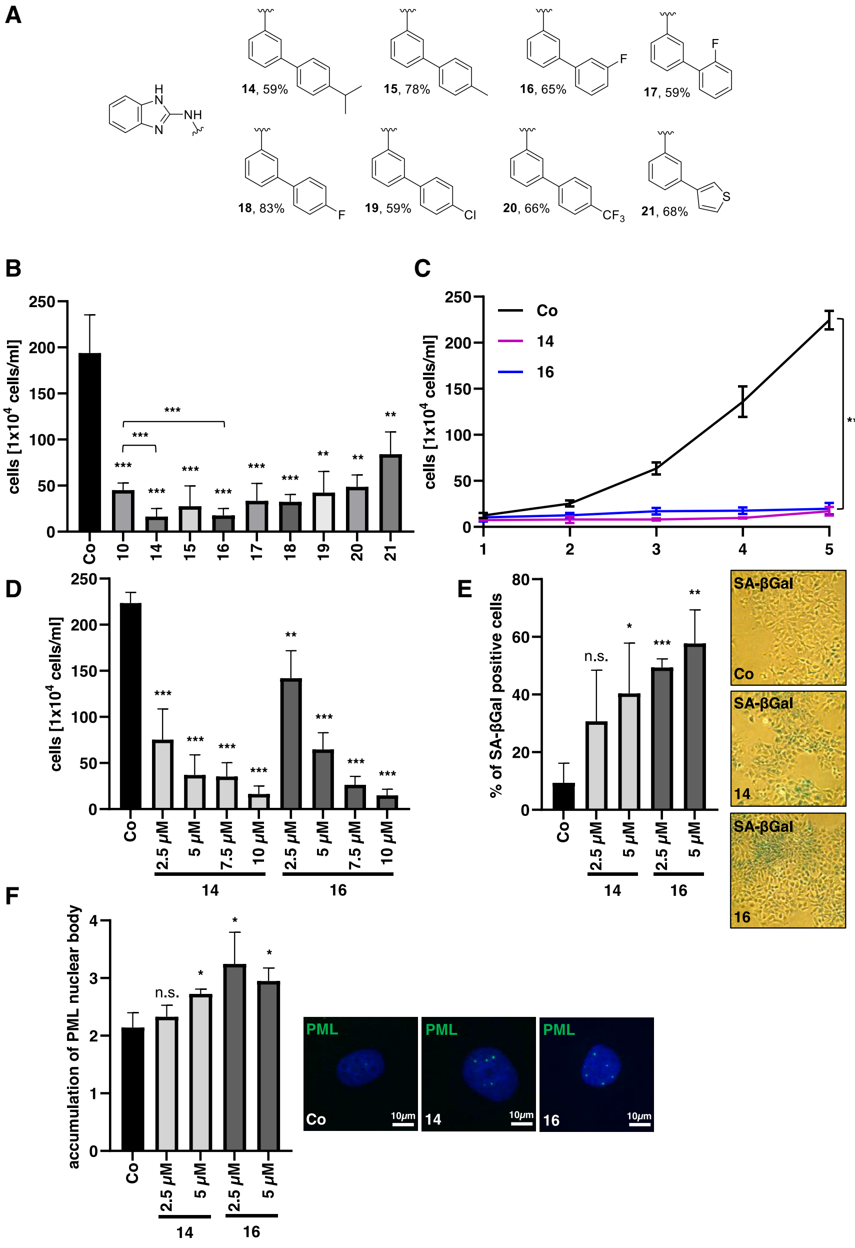

Figure 6. Constructing novel inhibitors of MRTF/SRF activity based on compound 10

(A) Structures of compounds **14**–**21**. The synthesis was performed according to Figure 5D. (B) Proliferation rates in HuH7 cells treated for 5 days with 10 μM **10** and **14**–**21** and DMSO as a control group. Data are means ± SD (n = 3); **$p < 0.01$, ***$p < 0.001$. (C) Proliferation rates in HuH7 cells treated with 10 μM **14** and **16** and DMSO as a control group. Data are means ± SD (n = 3); ***$p < 0.001$. (D) Concentration dependencies for inhibition of HuH7 cell proliferation upon administration of 2.5, 5, 7.5, and 10 μM compounds **14** and **16**. Data are means ± SD (n = 3); **$p < 0.01$, ***$p < 0.001$. (E) Quantification of SA-β-Gal-positive HuH7 cells treated with compounds **14** and **16** as indicated and DMSO as a control (left). β-Gal-positive cells were counted in 100 cells per condition. All data are means ± SD (n = 3); *$p < 0.05$, **$p < 0.01$, ***$p < 0.001$, n.s. not significant. Representative pictures of SA-β-gal staining with **14** and **16** (5 μM) (right). (F) Immunofluorescence staining with anti-PML antibody and DAPI for nuclear counterstaining in HuH7 cells treated with **14** and **16** and DMSO as a control. Quantification of PML nuclear body accumulation in 100 cells per condition. Scale bar, 10 μm. Data are means ± SD (n = 3); *$p < 0.05$, n.s. not significant.

tetraline core of NS8593 in direction A is tolerated better than inhibitors **2**–**4** and **6** with substituents pointing in direction B.

These findings opened up the possibility that a replacement of the tetraline core by a 3-biphenyl unit may be harnessed as a novel molecularly targeted therapeutic strategy. Indeed, we proved the efficacy of novel compounds **14** and **16** to inhibit SRF activity at 2.5 μM concentration. Our data on the biphenyls suggest that some degree of lipophilicity on the aromatic rings is crucial for activity, possibly to facilitate cell permeability. Within our series of 21 novel compounds, we provide first evidence that there is a clear correlation between inhibition of SRF reporter gene activity, HCC cell proliferation, and senescence induction, which can be exploited therapeutically. However, it has been shown that accumulation of senescent cells can lead to tissue damage and may drive age-related diseases.[10] Eliminating senescent cells provides a tissue-regenerative effect and prevents age-related disorders.[35,36] Therefore, a combined therapy of MRTF inhibitors and senolytics, potent novel pharmacological agents with selective cytotoxic activity on senescent cells that have recently been successful in preclinical studies, may be better tolerated by patients.[37,38] In a recent paper, it was shown that senolytics such as navitoclax or dasatinib eliminate senescent cells and prevent HCC progression.[39] An in-depth examination of targeting the TRPM7-MRTF axis in combination with senolytics will be the subject of future work. We envisage that these novel highly potent MRTF inhibitors combined with senolytics will have enormous potential to inhibit HCC cell proliferation by inducing cellular senescence and subsequent clearance of senescent cells.

MATERIALS AND METHODS

Synthesis

Compounds **1**–**8** were prepared via reductive amination from 2-aminobenzimidazole and the respective ketones (Figure 1A). Titanium(IV) isopropoxide and sodium triacetoxyborohydride were used as reagents and gave the desired compounds **1**–**8** in isolated

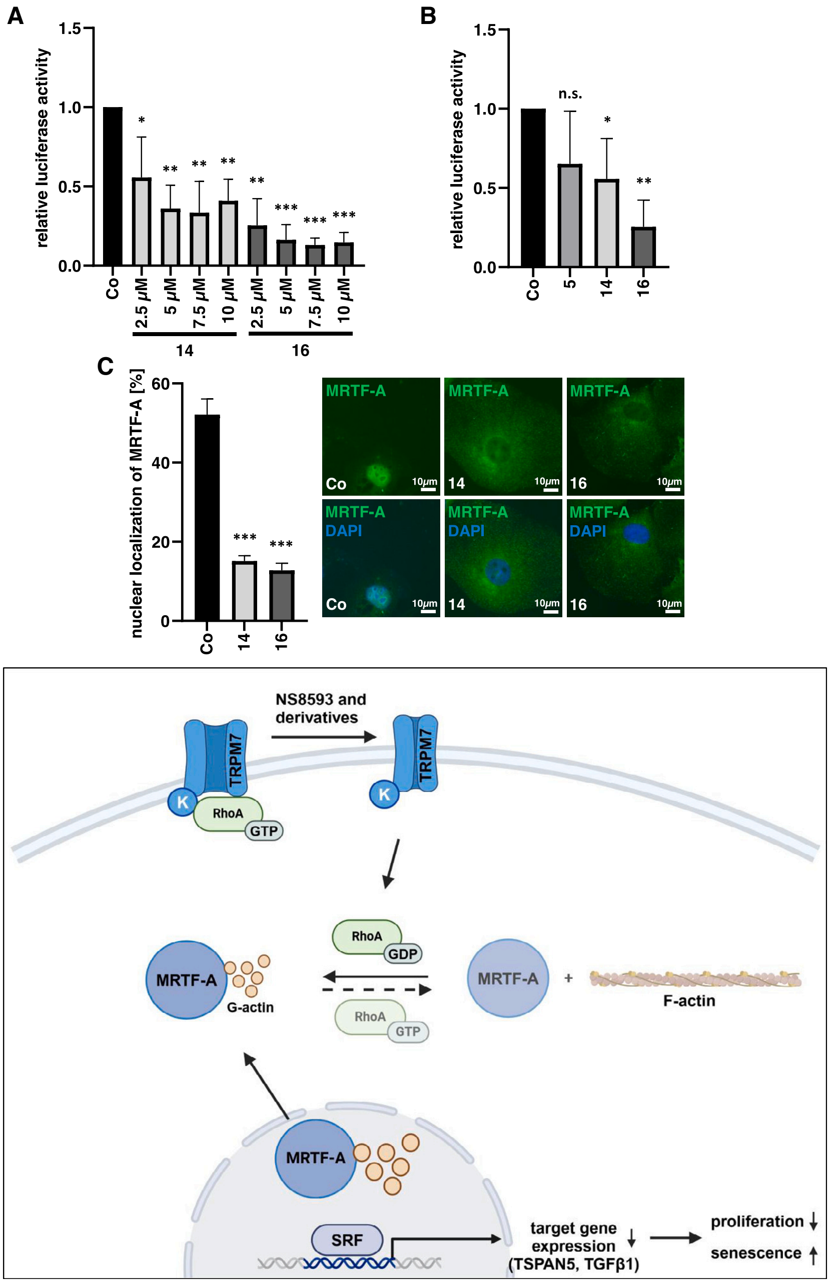

Figure 7. Compounds 14 and 16 inhibit MRTF/SRF activity

(A) HuH7 cells expressing 5×SRE and pRL-SV40P were treated with compounds **14** and **16** or DMSO as a control, and 24 h later, luciferase assays were performed for firefly luciferase and normalized to *Renilla* luciferase. Data are means ± SD ($n = 3$); *$p < 0.05$, **$p < 0.01$, ***$p < 0.001$. (B) HuH7 cells were treated as above. A concentration of 2.5 μM of **5**, **14**, and **16** was used. Data are means ± SD ($n = 3$); *$p < 0.05$, **$p < 0.01$, n.s. not significant. (C) Immunofluorescence staining with anti-MRTF-A antibody and DAPI for nuclear counterstaining in HuH7 cells treated for 18 h with 5 μM **14** and **16** and DMSO as a control group. Scale bar, 10 μm. Data are means ± SD ($n = 3$); ***$p < 0.001$. (D) The model for MRTF/SRF inhibition by NS and derivatives. TRPM7 blockade inhibits RhoA activation, which leads to disassembly of actin stress fibers and an increase in G-actin levels. Export of G-actin-bound MRTF-A reduces the amount of MRTF-A available to bind SRF and activate transcription of target genes such as TSPAN5 and TGF-β1, thereby reducing proliferation and inducing senescence.

yields ranging from 34%–58%. The 2- and 3-aminobiphenyl derivatives **9**–**13**, shown in Figures 5D and 6A, were accessible via nucleophilic aromatic substitution of 2-chlorobenzimidazole by the respective 2- or 3-aminobiphenyls in good to high yields (49%–87%) using microwave irradiation.

Docking studies

For docking studies, the cryo-EM structure of TRPM7 in complex with NS8593 (PDB: 8SIA)[24] was used. Docking was performed using Chimera (v.1.15)[40] with the AutoDockVina extension (v.1.2.0).[41] Visualization was performed with the PyMOL Molecular Graphics System v.1.3 (Schrödinger).[42]

Cell culture and transfection

HuH7 cells were cultured in RPMI 1640 medium (Sigma-Aldrich, Taufkirchen, Germany) and HuH6 and HEK293T cells in DMEM (Sigma-Aldrich). The medium was supplemented with 10% fetal bovine serum (Invitrogen, Karlsruhe, Germany) and 1% penicillin/streptomycin (Sigma-Aldrich). Lipofectamine 2000 (Invitrogen) was used for transient transfection of plasmids.

Cell proliferation assay

Cells were seeded and counted every 24 h for 5 days by using a Neubauer counting chamber.

Immunoblotting

Proteins were denatured by boiling in Laemmli buffer at 95°C. According to their molecular weight, the proteins were separated by SDS-PAGE for 2 h at 100 V using the Bio-Rad Western Blot System (Bio-Rad, Hercules, California, USA) and the power supply peQPOWER 300 (Peqlab Biotechnology, Erlangen, Germany). The proteins were then transferred onto a polyvinylidene fluoride membrane (Merck, Darmstadt, Germany), which was activated in 100% methanol and equilibrated in a transfer buffer. The transfer was done at 350 mA for approximately 2 h. To avoid non-specific protein binding, the membrane was blocked with 5% milk powder in TBS-T. Afterward, the membrane was incubated over night at 4°C with the primary (Table S2) and the respective secondary antibodies for 1 h at room temperature (Table S3). Both was done with gentle

shaking. The proteins were detected via chemiluminescence in a luminescent imager (ChemiDoc Imaging System, Bio-Rad).

SA-β-Gal staining

Cellular senescence of treated cells was determined using the Senescence-β-galactosidase staining kit according to the manufacturer's instructions (Cell Signaling Technology, Danvers, MA, USA). 300 cells were counted. The ratio stained to unstained cells were used to calculate the percentage of SA-β-gal-positive cells.

In situ proximity ligation assay

The DuoLink *In situ* Red Starter Kit Mouse/Rabbit (Sigma-Aldrich, Merck) was used according to the manufacturer's protocol for DuoLink *In Situ* Solutions (Sigma-Aldrich, Merck). Anti-TRPM7/TRPM6 (Abcam, Cambridge, UK), anti-RhoA (NewEast Bioscience, Malvern, PA, USA), anti-MRTF-A (Santa Cruz Biotechnology, Santa Cruz, CA, USA), and anti-Filamin A (Thermo Fisher Scientific, Schwerte, Germany) antibodies were used as primary antibodies for incubation (Table S4). Images were recorded with a fluorescence microscope (Nikon, Düsseldorf, Germany).

Immunofluorescence

Cells were fixated by a 10-min incubation with 4% paraformaldehyde in PBS. To permeabilize the cells, 0.2% Triton X-100 in PBS was used. Blocking was done with 1% BSA in PBS for 30 min at 37°C, followed by incubation with the respective primary antibodies (Table S4) for 1 h at room temperature and the secondary Alexa Fluor 488-coupled antibody (Invitrogen) (Table S5). The nucleus was stained with DAPI (Sigma-Aldrich). Images were taken using a fluorescence microscope (Nikon).

Invasion assay

The cell invasion assay was performed using Corning BioCoat Matrigel Invasion Chambers (Corning, Tewksbury, MA, USA) according to the manufacturer's instructions. 2.5×10^4 cells per well were seeded and incubated for 20 h at 37°C. The invaded cells were fixed with methanol, stained with crystal violet, and counted under a light microscope (Carl Zeiss, Oberkochen, Germany).

Luciferase assay

HuH7 cells were seeded in a 12-well plate and transiently transfected with 0.25 μg 5×SRE and 0.15 μg *Renilla* luciferase simian virus 40 (SV40) promoter reporter as an internal control. The next day, cells were treated with inhibitors, as indicated in the figure legends, and the Dual-Luciferase Reporter Assay System (Promega, Madison, Wisconsin, USA) and the BioFix Lumi-10 (Macherey-Nagel, Düren, Germany) were used. The firefly luciferase activity was normalized to the *Renilla* luciferase activity to compensate for divergent transfection efficiencies.

FLIP assay

HuH7 cells were plated on 35-mm cell culture dishes and transfected with 200 ng MRTF-A-GFP and 800 ng pEF-FLAG plasmid DNA by using JetPrime (Polyplus, Illkirch, France). After treatment of the cells with the respective inhibitor, cells were imaged at 37°C, 5% CO_2 in an Okolab Bold Line Cage Incubator using a Carl Zeiss LSM 700 confocal microscope with Axio Imager M2 and W Plan-Apochromat 63×/1.0 dipping objective. The software ZEN 2012 was used. Imaging parameters were as follows: pinhole diameter 1, resolution 256 × 256, bit depth 12, speed 7, line average 1, and zoom 2. The cytoplasm was bleached continuously starting after 3 scans with 2-s intervals using 100% laser power (488 nm/10 mW), and the nuclear fluorescence was recorded. The pre-bleach values were set to 1.

Aequorin-based Ca^{2+} influx assay

HEK293T cells were transfected with 2 μg TRPM7 plasmid DNA and 0.1 μg pG5A plasmid DNA. After 24 h, the cells were washed with Mg^{2+}-free HEPES-buffered saline (HBS) containing 140 mM NaCl, 5.4 mM KCl, 0.5 mM $CaCl_2$, 5 mM HEPES (pH 7.4), and 10 mM glucose and resuspended in Mg^{2+}-free HBS. After 30 min of incubation with 5 μM coelenterazine (Biaffin) at room temperature to reconstitute the aequorin, cells were washed two times by centrifugation at 2,000 rpm for 5 min (Heraeus Pico 17 microcentrifuge, Thermo Fisher Scientific). Then, they were resuspended in Mg^{2+}-free HBS and plated into 96-well plates. Luminescence was detected at room temperature using a CLARIOstar microplate reader (BMG Labtech). $CaCl_2$-containing Mg^{2+}-free HBS was injected in the presence or absence of the inhibitors to increase the extracellular Ca^{2+} concentration to 5 mM. Cell lysis with 0.1% (v/v) Triton X-100 in the Mg^{2+}-free HBS was used to terminate the experiment, and total bioluminescence was measured. The analysis of the bioluminescence rates (counts/s) was done in 1-s intervals and calibrated as $[Ca^{2+}]_i$ values using the following equation:

$$p[Ca^{2+}]_i = 0,332588\,(-\log(k)) + 5,5593$$

k represents the rate of aequorin consumption.

The following equation was used to determine the IC_{50} values of the inhibitors:

$$E(c) = E_{min} + \frac{E_{max} + E_{min}}{1 + \frac{c^h}{{IC_{50}}^h}}$$

E(c) is the functional effect at the concentration c of the inhibitor, E_{min} is the minimal effect, E_{max} is the maximal effect, IC_{50} is the concentration of the inhibitor that produces the half-maximal effect, and h is the Hill coefficient. GraphPad Prism 8.4.0 was used for fitting.

Statistical analysis

Statistical analysis was carried out using Student's t test or Mann-Whitney test. Data from three independent experiments were analyzed and values presented as mean ± SD unless otherwise indicated (Table S6). Values are considered statistically significant with $^*p < 0.05$, $^{**}p < 0.01$, and $^{***}p < 0.001$.

DATA AND CODE AVAILABILITY

The data presented in this study are available in this article and supplemental information.

SUPPLEMENTAL INFORMATION

Supplemental information can be found online at https://doi.org/10.1016/j.omton.2024.200855.

ACKNOWLEDGMENTS

This work was funded by MU2737/2-4, HE5413/9-4, and GRK1910 of the Deutsche Forschungsgemeinschaft. M.K.V. was supported by Research Council of Finland grant 338281, and V.C. and T.G. by DFG TRR 152 (P15) and GRK 2338 RTG (P10). We thank Ron Prywes (Columbia University, New York) for reviewing and editing the manuscript and Joanna Zaisserer and Anna Erbacher (Walther-Straub Institute, LMU Munich) for technical assistance. BioRender was used for graphical illustrations.

AUTHOR CONTRIBUTIONS

Conceptualization, S.M. and M.R.H.; methodology, M.J.F., P. Wenisch, P. Wohlleben, L.R., V.C., and S.K.; supervision, S.M., M.R.H., M.K.V., and T.G.; formal analysis, M.J.F.; funding acquisition, S.M. and M.R.H; writing, S.M.

DECLARATION OF INTERESTS

The authors declare no competing interests.

REFERENCES

1. Sung, H., Ferlay, J., Siegel, R.L., Laversanne, M., Soerjomataram, I., Jemal, A., and Bray, F. (2021). Global Cancer Statistics 2020: GLOBOCAN Estimates of Incidence and Mortality Worldwide for 36 Cancers in 185 Countries. CA. Cancer J. Clin. *71*, 209–249.
2. El-Serag, H.B. (2007). Epidemiology of hepatocellular carcinoma in USA. Hepatol. Res. *37*, S88–S94.
3. Whittaker, S., Marais, R., and Zhu, A.X. (2010). The role of signaling pathways in the development and treatment of hepatocellular carcinoma. Oncogene *29*, 4989–5005.
4. Allemani, C., Matsuda, T., Di Carlo, V., Harewood, R., Matz, M., Nikšić, M., Bonaventure, A., Valkov, M., Johnson, C.J., Estève, J., et al. (2018). Global surveillance of trends in cancer survival 2000–14 (CONCORD-3): analysis of individual records for 37 513 025 patients diagnosed with one of 18 cancers from 322 population-based registries in 71 countries. Lancet *391*, 1023–1075.
5. Hampl, V., Martin, C., Aigner, A., Hoebel, S., Singer, S., Frank, N., Sarikas, A., Ebert, O., Prywes, R., Gudermann, T., and Muehlich, S. (2013). Depletion of the transcriptional coactivators megakaryoblastic leukaemia 1 and 2 abolishes hepatocellular carcinoma xenograft growth by inducing oncogene-induced senescence. EMBO Mol. Med. *5*, 1367–1382.
6. Ohrnberger, S., Thavamani, A., Braeuning, A., Lipka, D.B., Kirilov, M., Geffers, R., Autenrieth, S.E., Römer, M., Zell, A., Bonin, M., et al. (2015). Dysregulated serum response factor triggers formation of hepatocellular carcinoma. Hepatology *61*, 979–989.
7. Hermanns, C., Hampl, V., Holzer, K., Aigner, A., Penkava, J., Frank, N., Martin, D.E., Maier, K.C., Waldburger, N., Roessler, S., et al. (2017). The novel MKL target gene myoferlin modulates expansion and senescence of hepatocellular carcinoma. Oncogene *36*, 3464–3476.
8. Zhang, L., Li, H.-L., Zhang, D.-D., and Cui, X.-C. (2021). Therapeutic effects of myocardin-related transcription factor A (MRTF-A) knockout on experimental mice with nonalcoholic steatohepatitis induced by high-fat diet. Hum. Exp. Toxicol. *40*, 1634–1645.
9. Shi, Z., Ren, M., and Rockey, D.C. (2020). Myocardin and myocardin-related transcription factor-A synergistically mediate actin cytoskeletal-dependent inhibition of liver fibrogenesis. Am. J. Physiol. Gastrointest. Liver Physiol. *318*, G504–G517.
10. Mittermeier, C., Konopa, A., and Muehlich, S. (2020). Molecular Mechanisms to Target Cellular Senescence in Hepatocellular Carcinoma. Cells *9*, 2540.
11. Voringer, S., Schreyer, L., Nadolni, W., Meier, M.A., Woerther, K., Mittermeier, C., Ferioli, S., Singer, S., Holzer, K., Zierler, S., et al. (2020). Inhibition of TRPM7 blocks MRTF/SRF-dependent transcriptional and tumorigenic activity. Oncogene *39*, 2328–2344.
12. Nadler, M.J., Hermosura, M.C., Inabe, K., Perraud, A.-L., Zhu, Q., Stokes, A.J., Kurosaki, T., Kinet, J.-P., Penner, R., Scharenberg, A.M., and Fleig, A. (2001). LTRPC7 is a Mg·ATP-regulated divalent cation channel required for cell viability. Nature *411*, 590–595.
13. Schmitz, C., Perraud, A.-L., Johnson, C.O., Inabe, K., Smith, M.K., Penner, R., Kurosaki, T., Fleig, A., and Scharenberg, A.M. (2003). Regulation of Vertebrate Cellular Mg^{2+} Homeostasis by TRPM7. Cell *114*, 191–200.
14. Ryazanova, L.V., Dorovkov, M.V., Ansari, A., and Ryazanov, A.G. (2004). Characterization of the Protein Kinase Activity of TRPM7/ChaK1, a Protein Kinase Fused to the Transient Receptor Potential Ion Channel. J. Biol. Chem. *279*, 3708–3716.
15. Matsushita, M., Kozak, J.A., Shimizu, Y., McLachlin, D.T., Yamaguchi, H., Wei, F.-Y., Tomizawa, K., Matsui, H., Chait, B.T., Cahalan, M.D., and Nairn, A.C. (2005). Channel Function Is Dissociated from the Intrinsic Kinase Activity and Autophosphorylation of TRPM7/ChaK1. J. Biol. Chem. *280*, 20793–20803.
16. Mittermeier, L., Demirkhanyan, L., Stadlbauer, B., Breit, A., Recordati, C., Hilgendorff, A., Matsushita, M., Braun, A., Simmons, D.G., Zakharian, E., et al. (2019). TRPM7 is the central gatekeeper of intestinal mineral absorption essential for postnatal survival. Proc. Natl. Acad. Sci. USA *116*, 4706–4715.
17. Chubanov, V., Köttgen, M., Touyz, R.M., and Gudermann, T. (2024). TRPM channels in health and disease. Nat. Rev. Nephrol. *20*, 175–187.
18. Yee, N.S. (2017). Role of TRPM7 in Cancer: Potential as Molecular Biomarker and Therapeutic Target. Pharmaceuticals *10*, 39.
19. Chubanov, V., Mederos y Schnitzler, M., Meißner, M., Schäfer, S., Abstiens, K., Hofmann, T., and Gudermann, T. (2012). Natural and synthetic modulators of SK (Kca2) potassium channels inhibit magnesium-dependent activity of the kinase-coupled cation channel TRPM7. Br. J. Pharmacol. *166*, 1357–1376.
20. Habermann, E. (1984). Apamin. Pharmacol. Ther. *25*, 255–270.
21. Simó-Vicens, R., Kirchhoff, J.E., Dolce, B., Abildgaard, L., Speerschneider, T., Sørensen, U.S., Grunnet, M., Diness, J.G., and Bentzen, B.H. (2017). A new negative allosteric modulator, AP14145, for the study of small conductance calcium-activated potassium (KCa2) channels. Br. J. Pharmacol. *174*, 4396–4408.
22. Sørensen, U.S., Strøbaek, D., Christophersen, P., Hougaard, C., Jensen, M.L., Nielsen, E.Ø., Peters, D., and Teuber, L. (2008). Synthesis and Structure−Activity Relationship Studies of 2-(N-Substituted)-aminobenzimidazoles as Potent Negative Gating Modulators of Small Conductance Ca2+-Activated K+ Channels. J. Med. Chem. *51*, 7625–7634.
23. Schreyer, L., Mittermeier, C., Franz, M.J., Meier, M.A., Martin, D.E., Maier, K.C., Huebner, K., Schneider-Stock, R., Singer, S., Holzer, K., et al. (2021). Tetraspanin 5 (TSPAN5), a Novel Gatekeeper of the Tumor Suppressor DLC1 and Myocardin-Related Transcription Factors (MRTFs), Controls HCC Growth and Senescence. Cancers *13*, 5373.
24. Nadezhdin, K.D., Correia, L., Narangoda, C., Patel, D.S., Neuberger, A., Gudermann, T., Kurnikova, M.G., Chubanov, V., and Sobolevsky, A.I. (2023). Structural mechanisms of TRPM7 activation and inhibition. Nat. Commun. *14*, 2639.
25. Pratsch, G., Wallaschkowski, T., and Heinrich, M.R. (2012). The Gomberg-Bachmann reaction for the arylation of anilines with aryl diazotates. Chemistry *18*, 11555–11559.
26. Liu, Q.-P., Hou, X.-D., Li, N., and Zong, M.-H. (2012). Ionic liquids from renewable biomaterials: synthesis, characterization and application in the pretreatment of biomass. Green Chem. *14*, 304–307.
27. Graßl, F., Bock, L., Huete-Huerta González, Á., Schiller, M., Gmeiner, P., König, J., Fromm, M.F., Hübner, H., and Heinrich, M.R. (2023). Exploring Structural

Determinants of Bias among D4 Subtype-Selective Dopamine Receptor Agonists. J. Med. Chem. *66*, 9710–9730.

28. Muehlich, S., Wang, R., Lee, S.-M., Lewis, T.C., Dai, C., and Prywes, R. (2008). Serum-Induced Phosphorylation of the Serum Response Factor Coactivator MKL1 by the Extracellular Signal-Regulated Kinase 1/2 Pathway Inhibits Its Nuclear Localization. Mol. Cell Biol. *28*, 6302–6313.
29. Vartiainen, M.K., Guettler, S., Larijani, B., and Treisman, R. (2007). Nuclear Actin Regulates Dynamic Subcellular Localization and Activity of the SRF Cofactor MAL. Science *316*, 1749–1752.
30. Hyrskyluoto, A., and Vartiainen, M.K. (2020). Regulation of nuclear actin dynamics in development and disease. Curr. Opin. Cell Biol. *64*, 18–24.
31. Lundquist, M.R., Storaska, A.J., Liu, T.-C., Larsen, S.D., Evans, T., Neubig, R.R., and Jaffrey, S.R. (2014). Redox Modification of Nuclear Actin by MICAL-2 Regulates SRF Signaling. Cell *156*, 563–576.
32. Kollewe, A., Chubanov, V., Tseung, F.T., Correia, L., Schmidt, E., Rössig, A., Zierler, S., Haupt, A., Müller, C.S., Bildl, W., et al. (2021). The molecular appearance of native TRPM7 channel complexes identified by high-resolution proteomics. Elife *10*, e68544.
33. Romagnani, A., Vettore, V., Rezzonico-Jost, T., Hampe, S., Rottoli, E., Nadolni, W., Perotti, M., Meier, M.A., Hermanns, C., Geiger, S., et al. (2017). TRPM7 kinase activity is essential for T cell colonization and alloreactivity in the gut. Nat. Commun. *8*, 1917.
34. Russ, A.P., and Lampel, S. (2005). The druggable genome: an update. Drug Discov. Today *10*, 1607–1610.
35. Eggert, T., Wolter, K., Ji, J., Ma, C., Yevsa, T., Klotz, S., Medina-Echeverz, J., Longerich, T., Forgues, M., Reisinger, F., et al. (2016). Distinct Functions of Senescence-Associated Immune Responses in Liver Tumor Surveillance and Tumor Progression. Cancer Cell *30*, 533–547.
36. Baker, D.J., Wijshake, T., Tchkonia, T., LeBrasseur, N.K., Childs, B.G., van de Sluis, B., Kirkland, J.L., and van Deursen, J.M. (2011). Clearance of p16Ink4a-positive senescent cells delays ageing-associated disorders. Nature *479*, 232–236.
37. Childs, B.G., Gluscevic, M., Baker, D.J., Laberge, R.-M., Marquess, D., Dananberg, J., and van Deursen, J.M. (2017). Senescent cells: an emerging target for diseases of ageing. Nat. Rev. Drug Discov. *16*, 718–735.
38. Fuhrmann-Stroissnigg, H., Ling, Y.Y., Zhao, J., McGowan, S.J., Zhu, Y., Brooks, R.W., Grassi, D., Gregg, S.Q., Stripay, J.L., Dorronsoro, A., et al. (2017). Identification of HSP90 inhibitors as a novel class of senolytics. Nat. Commun. *8*, 422.
39. Li, F., Huangyang, P., Burrows, M., Guo, K., Riscal, R., Godfrey, J., Lee, K.E., Lin, N., Lee, P., Blair, I.A., et al. (2020). FBP1 loss disrupts liver metabolism and promotes tumorigenesis through a hepatic stellate cell senescence secretome. Nat. Cell Biol. *22*, 728–739.
40. Pettersen, E.F., Goddard, T.D., Huang, C.C., Couch, G.S., Greenblatt, D.M., Meng, E.C., and Ferrin, T.E. (2004). UCSF Chimera—a visualization system for exploratory research and analysis. J. Comput. Chem. *25*, 1605–1612.
41. Trott, O., and Olson, A.J. (2010). AutoDock Vina: improving the speed and accuracy of docking with a new scoring function, efficient optimization, and multithreading. J. Comput. Chem. *31*, 455–461.
42. Schrodinger, L. (2015). The PyMOL molecular graphics system. Version *1*, 8.

OMTON, Volume *32*

Supplemental information

Identification of novel inhibitors of the transcriptional coactivator MRTF-A for HCC therapy

Miriam Jasmin Franz, Pia Wenisch, Petra Wohlleben, Laura Rupprecht, Vladimir Chubanov, Thomas Gudermann, Salla Kyheröinen, Maria Kristina Vartiainen, Markus R. Heinrich, and Susanne Muehlich

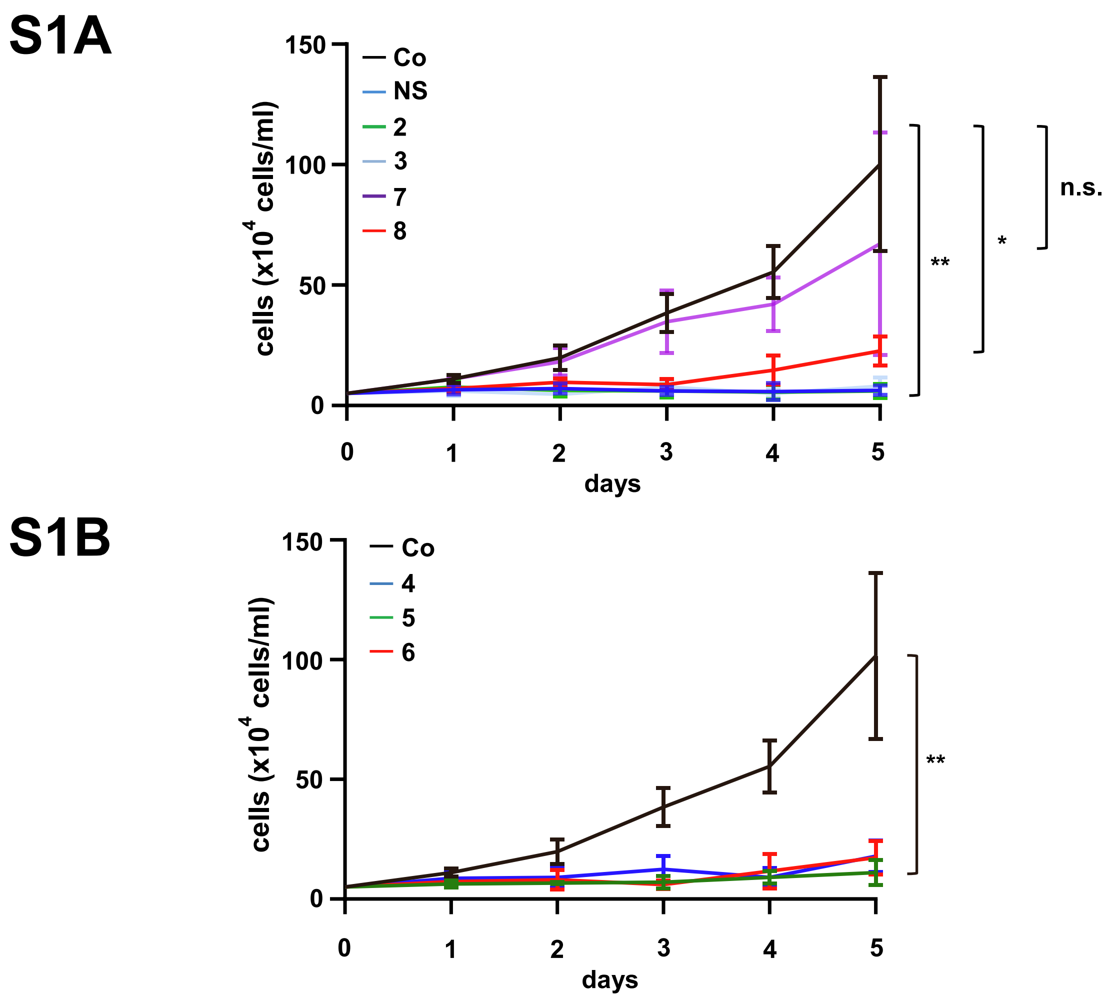

Figure S1: Inhibition of TRPM7 by NS8593 and analogues induces HCC proliferation arrest. Proliferation rates in HuH6 cells (A) treated with 30 µM NS8593, **2, 3, 7, 8** and DMSO (control, Co) and (B) inhibited with 10 µM **4**, **5**, **6** and DMSO (control, Co). Data are means ± SD (n=3).

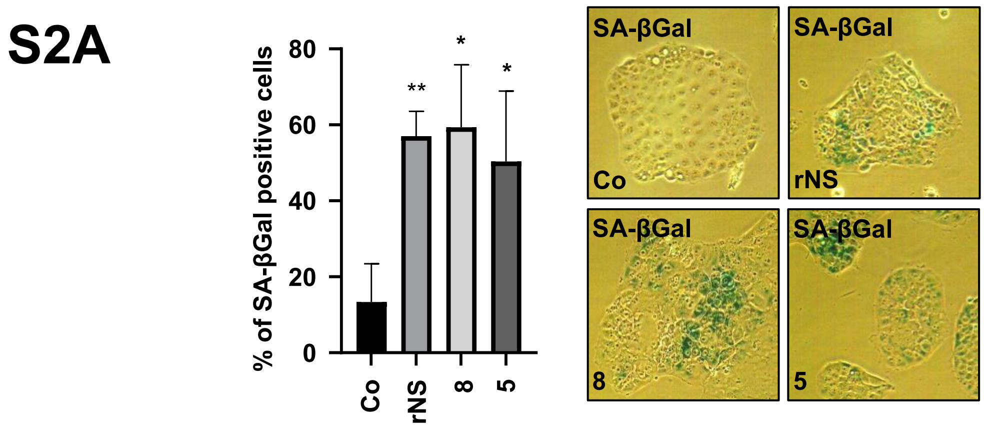

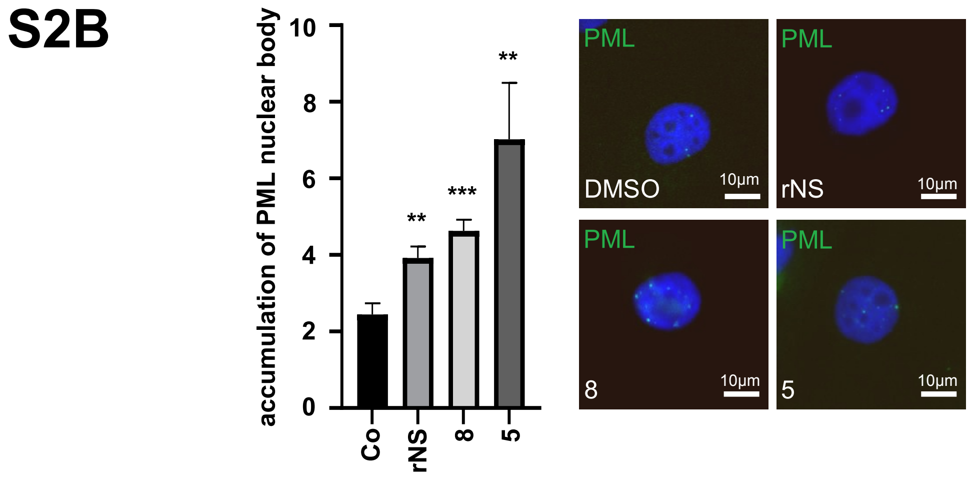

Figure S2: Inhibition of TRPM7 by NS8593 and analogues induces cellular senescence (A) Quantification of senescence-associated beta-galactosidase positive cells of HuH6 cells treated with 5 µM **5** and 30 µM rNS, **8** and DMSO (control, Co). Beta-Gal positive cells were counted in 100 cells per condition. All data are means ± SD (n = 3). (B) Immunofluorescence staining with anti-PML antibody and DAPI for nuclear counterstaining in HuH7 cells inhibited as in described in (A). Quantification of PML nuclear body accumulation by counting the green spots in 100 cells per condition. Data are means ± SD (n=3).

S3A

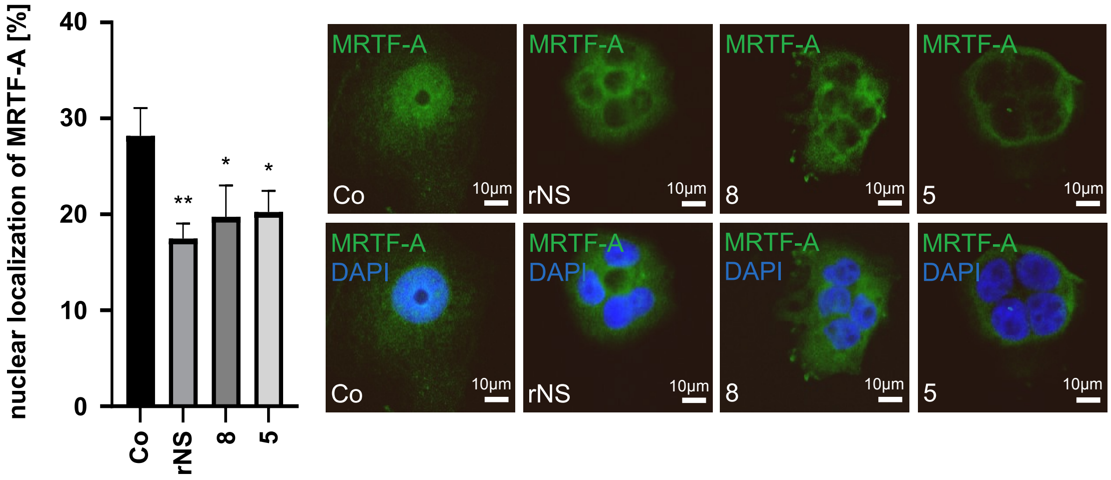

Figure S3: Enhanced MRTF-A nuclear export upon administration of rNS and compounds 5 and 8 (A) Immunofluorescence staining with anti-MRTF-A antibody and DAPI for nuclear counterstaining in HuH6 cells treated with 5 µM **5** and 30 µM rNS, **8** and DMSO (control, Co). Scale bar, 10 µm. Data are means ± SD (n=3).

S4A

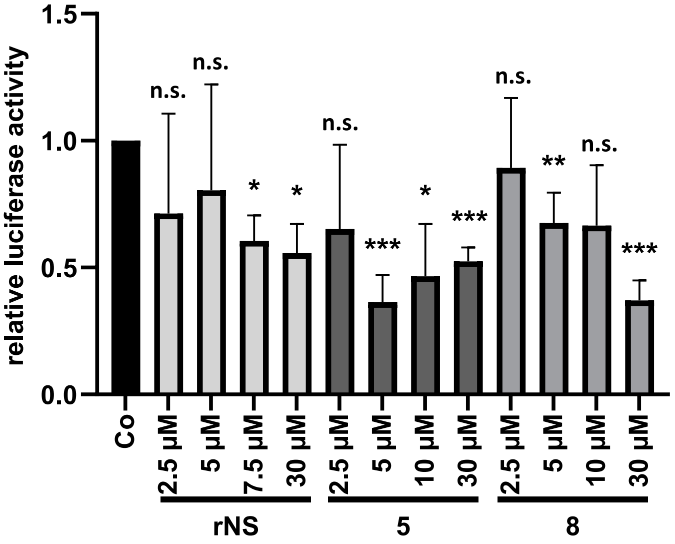

Figure S4A: Inhibitors 5 and 8 reduce SRF activity. (A) HuH7 cells expressing a SRE-dependent luciferase reporter gene (5xSRE) and a Renilla luciferase internal control (pRL-SV40P) were treated with rNS, **5**, **8** or DMSO (control, Co) as indicated and 24 h later luciferase assays performed for firefly luciferase and normalized to Renilla luciferase. Data are means ± SD (n=3).

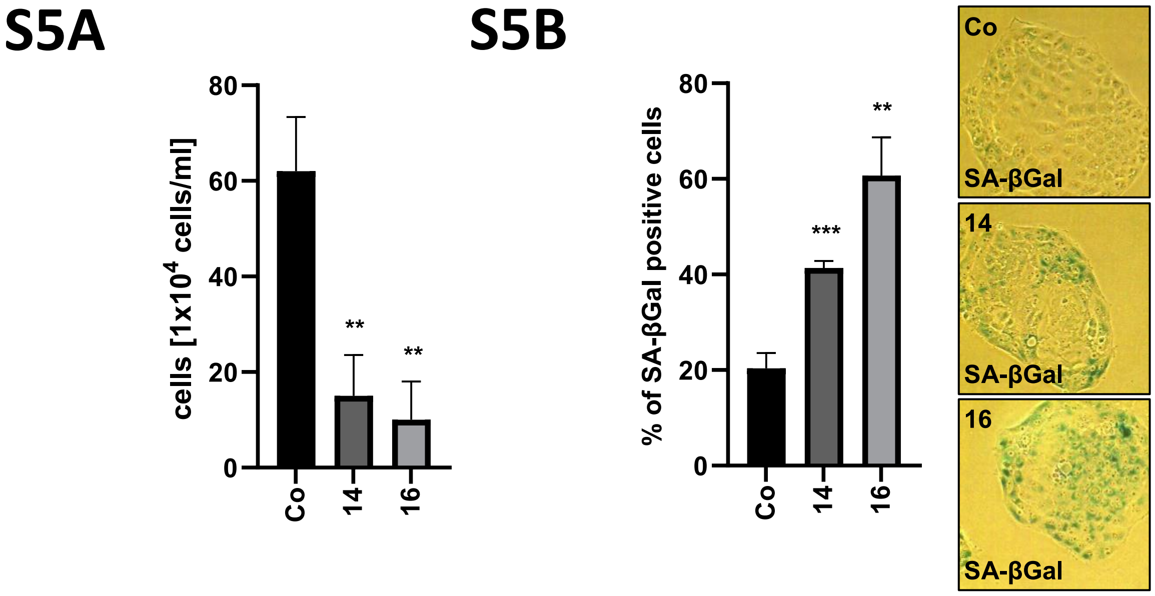

Figure S5: Novel inhibitors 14 and 16 inhibit HCC cell proliferation by inducing cellular senescence (A) Proliferation rates in HuH6 cells inhibited with 10 μM **14** and **16** and DMSO (control, Co). Data are means ± SD (n=3). (B) Quantification of senescence-associated beta-galactosidase positive HuH6 cells treated with 5 μM **14** and **16** and DMSO (control, Co). Beta-Gal positive cells were counted in 100 cells per condition. All data are means ± SD (*n* = 3).

Table S1: IC_{50} values and hill slopes for TRPM7 inhibition

derivative	IC_{50} (µM)	Hill slopes	n
NS8593	1.464	-1.432	3
1, rNS	1.761	-1.370	3
2	3.740	-1.165	3
3	5.739	-1.224	3
4	2.798	-1.123	3
5	1.093	-1.448	3
6	1.688	-1.292	3
7	63.31	-0.9579	3
8	2.817	-1.210	3

Table S2: Primary antibodies used for immunoblotting

antibody	manufacturer
anti-HSP90 (mouse)	Santa Cruz Biotechnology, Inc., Dallas, TX, USA
anti-TGFβ1 (mouse)	Santa Cruz Biotechnology, Inc., Dallas, TX, USA
anti-TSPAN5 (rabbit)	Merck, Darmstadt, Germany

Table S3: Secondary antibodies used for immunoblotting

antibody	manufacturer
anti-mouse IgG, HRP-linked antibody	Cell Signaling Technology, Danvers, MA, USA
anti-rabbit IgG, HRP-linked antibody	Cell Signaling Technology, Danvers, MA, USA

Table S4: Primary antibodies used for immunofluorescence and proximity ligation assay

antibody	manufacturer
anti-MRTF-A (mouse)	Santa Cruz Biotechnology, Inc., Dallas, TX, USA
anti-TRPM7+TRPM6 (rabbit)	Abcam, Cambridge, UK
anti-RhoA (mouse)	NewEast Bioscience, Malvern, PA, USA

Table S5: Secondary antibodies used for immunofluorescence

antibody	manufacturer
Donkey anti-mouse IgG, Alexa-488-linked antibody	Invitrogen, Karlsruhe, Germany

Table S6: p-values

Figure	Compared samples	p-values
**1D**	Co vs. 2.5 µM 5	0.0474
	Co vs. 5 µM 5	0.0016
	Co vs. 7.5 µM 5	0.0014
	Co vs. 10 µM 5	0.0014
**1E**	Co vs. NS8593	0.0016
	Co vs. 4 / 5 / 6	< 0.001

**1F**	Co vs. 7	0.0296
	Co vs. NS8593 / rNS /2 / 3 / 8	< 0.001
**2A**	Co vs. 5 µM 5	0.0073
	Co vs. 7.5 µM 5	0.0246
	Co vs. 10 µM 5	0.0034
**2B**	Co vs. 20 µM 8	0.0017
	Co vs. 30 µM 8	0.0011
**2C**	Co vs. 20 µM rNS	0.0049
	Co vs. 30 µM rNS	< 0.001
**2D**	Co vs. rNS	0.0082
	Co vs. 8	0.0014
	Co vs. 5	0.0032
**2E**	Co vs. rNS	0.0013
	Co vs. 8	< 0.001
	Co vs. 5	0.0031
**3A**	Co vs. rNS	0.031
	Co vs. 8 / 5	< 0.001
**3B**	Co vs. rNS	0.0019
	Co vs. 8 / 5	< 0.001
	rNS vs. 8	0.0427
	rNS vs. 5	0.0113
**3D**	Co vs. NS / 5	< 0.001
**4A**	Co vs. rNS / 5 / 8	< 0.001
**4B**	Co vs. 8	0.0094
	Co vs. 5	< 0.001
**4C TSPAN5**	Co vs. 5 µM 5	< 0.001
	Co vs. 7.5 µM 5	0.0326
	Co vs. 10 µM 5	0.0015
**4C TGFβ1**	Co vs. 5 µM 5	0.0075
	Co vs. 7.5 µM 5	< 0.001
	Co vs. 10 µM 5	< 0.001
**4D**	Co vs. 20 µM 8	0.012
	Co vs. 30 µM 8	0.0078
	Co vs. 40 µM 8	0.0068
	Co vs. 50 µM 8	0.0071
**5E**	Co vs. 9	0.0076
	Co vs. 10 / 12	< 0.001
**5F**	Co vs. 10	< 0.001
**6B**	Co vs. 10 / 14 / 15 / 16 / 17 / 18	< 0.001
	Co vs. 19	0.0013
	Co vs. 20	0.0012
	Co vs. 21	0.0063
	10 vs. 14 / 16	< 0.001
**6C**	Co vs. 14 / 16	< 0.001
**6D**	Co vs. 2.5µM-10µM 14	< 0.001
	Co vs. 2.5 µM 16	0.0037

	Co vs. 5µM-10µM 16	< 0.001
**6E**	Co vs. 5 µM 14	0.0458
	Co vs. 2.5 µM 16	< 0.001
	Co vs. 5 µM 16	0.0035
**7A**	Co vs. 2.5 µM 14	0.0321
	Co vs. 5 µM 14	0.0017
	Co vs. 7.5 µM 14	0.0043
	Co vs. 10 µM 14	0.0017
	Co vs. 2.5 µM 16	0.0016
	Co vs. 5µM-10µM 16	< 0.001
**7B**	Co vs. 14	0.0321
	Co vs. 16	0.0016
**7C**	Co vs. 14 / 16	< 0.001
